# A molecular assembler that produces polymers

**DOI:** 10.1038/s41467-020-17814-0

**Published:** 2020-08-19

**Authors:** Anthonius H. J. Engwerda, Stephen P. Fletcher

**Affiliations:** Department of Chemistry, Chemistry Research Laboratory University of Oxford, 12 Mansfield Road, Oxford, OX1 3TA UK

**Keywords:** Origin of life, Synthetic chemistry methodology, Self-assembly

## Abstract

Molecular nanotechnology is a rapidly developing field, and tremendous progress has been made in developing synthetic molecular machines. One long-sought after nanotechnology is systems able to achieve the assembly-line like production of molecules. Here we report the discovery of a rudimentary synthetic molecular assembler that produces polymers. The molecular assembler is a supramolecular aggregate of bifunctional surfactants produced by the reaction of two phase-separated reactants. Initially self-reproduction of the bifunctional surfactants is observed, but once it reaches a critical concentration the assembler starts to produce polymers instead of supramolecular aggregates. The polymer size can be controlled by adjusting temperature, reaction time, or introducing a capping agent. There has been considerable debate about molecular assemblers in the context of nanotechnology, our demonstration that primitive assemblers may arise from simple phase separated reactants may provide a new direction for the design of functional supramolecular systems.

## Abstract (web summary)

The feasibility of molecular assemblers as a device to control chemical reactions by positioning molecules with atomic precision is a matter of debate in the literature. Here the authors describe of a rudimentary synthetic molecular assembler, supramolecular aggregate of bifunctional surfactants produced by the reaction of two phase-separated reactants that produces polymers.

## Introduction

Molecular nanotechnology is a rapidly developing field that has, for several decades now, explored the possibilities of miniaturizing technology found on the macroscopic scale. One area of nanotechnology that has made tremendous progress is developing molecular machines; assemblies that can undergo controlled directional movement driven by external stimuli^1,2^. Macroscopic devices often serve as inspiration for molecular analogs of hinges^3,4^, rotors^5–7^, switches^8^, and even complex structures such as cars^9,10^. Using molecular machines, functions may be performed^11^, such as transporting chemicals^12^, macroscopic movement^13–15^, and catalysis^16^. Although practically still at the proof of concept stage, the array of processes that can be catalyzed by molecular machines is impressive and includes polymerization^17,18^ and asymmetric synthesis^19,20^.

Although the field of molecular nanotechnology started to make experimental progress in the 1990’s, the basic concepts were introduced by Richard Feynman in his iconic there’s plenty of room at the bottom lecture^21^. Popularity later grew thanks to Eric Drexler, who introduced the concept of a molecular assembler to nanotechnology; a device able to guide chemical reactions by positioning reactive molecules with atomic precision (Note that the term assembler is also used in computer language, referring to a program that translates computer language into machine code)^22^. It was proposed that such an assembler would be able to create any desired molecule with high selectivity and efficiency. In order to produce substantial amounts of product, Drexler envisioned that the molecular assembler should be able to self-replicate. After an adequate amount of assembler has been formed, it could be re-programmed to produce the required product. Drexler’s vision of a molecular assembler later became the subject of a heated and well-publicized debate between Drexler and Richard Smalley, who questioned the feasibility of such an assembler^23^. Although it is widely accepted that the type of molecular assembler envisioned by Drexler cannot be created, some artificial systems come close to this concept^18^. In addition, nature’s interpretation of a molecular assembler can be found in the translation process of mRNA into a protein by the ribosome. Information encoded in the DNA of cells is transcribed to mRNA, to which the ribosome can attach. While moving along the mRNA strand, the ribosome translates the encoded information into a growing peptide chain. This is done by base-pairing the mRNA to tRNA fragments, which have the required amino acids attached to them. This results in a step-wise growth of the polypeptide chain with an error rate of only 10^−8^ ^24^. Although limited to polypeptides, this model of a molecular assembler can be used to produce any desired sequence of amino acids in high precision.

**Fig. 1:**
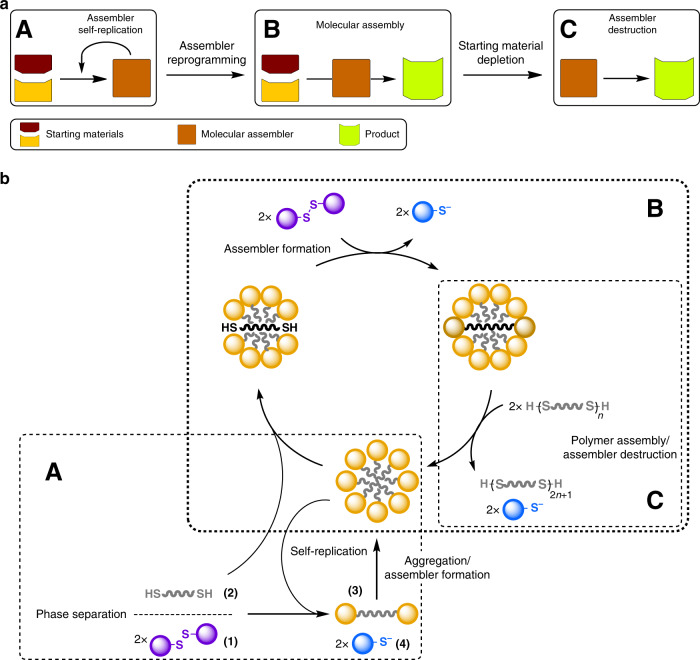
Lifecycle of the molecular assembler. **a** Schematic representation of three stages in the lifecycle of a possible design of the molecular assembler described here. (A) Initially, assembler self-replication is required to create substantial amounts of the molecular assembler. (B) Next, after reprogramming the assembler starts to produce the desired product. (C) When the assembler has completed its function, and the system is depleted of fuel or starting material, the assembler is destroyed to produce more product. **b** The three stages of the molecular assembler realized in our reported system. (**A**) Formation of the assembler: the reaction between the phase-separated dithiol and disulfide yields a (bola)amphiphilic compound that can self-assemble into supramolecular micelles. These micelles help solubilizing the apolar dithiol, thereby increasing the rate of formation of the amphiphile, a process known as physical autocatalysis. (**B**) Functional molecular assembler: the micelles keep solubilizing the apolar thiol, thereby generating more amphiphile. This amphiphile is then consumed in a second reaction with additional equivalents of thiol, resulting in the formation (and elongation) of polydisulfides. The assembler produces and consumes its building blocks at the same rate, resulting in a constant concentration of amphiphile. (**C**) Self-destruction of the assembler: when the starting dithiol is depleted, no more amphiphile can be formed. The assembler uses the remaining amphiphile to continue the polymerization process, thereby consuming its own building blocks until it has completely disappeared.

One possible interpretation of Drexler’s design of an assembler in terms of chemistry, is shown in Fig. 1 top, where the molecular assembler is an entity that forms from a chemical reaction. The assembler is required to self-replicate, and so this reaction could be autocatalytic. When substantial amounts of the assembler are formed, the assembler starts producing its intended product, by catalysing a second type of reaction. Upon completion of this function, the assembler could either go into a dormant state, or self-destroy.

During the course of our work on self-replicating and out-of-equilibrium supramolecular systems^25–27^, we discovered a system that we realized shares many features of the previously envisaged molecular assembler, that system is described here. Early in the system’s lifecycle, the reaction between two (phase-separated) reagents produces surfactants that self-assemble into functional supramolecular aggregates; the assembler. These aggregates self-replicate (via physical autocatalysis), resulting in an exponential increase in their concentration. However, instead of self-replicating until all available starting materials are consumed, at some concentration the assembler reaches a steady state. During this steady state, the assembler produces a new product (in this case polymers). After the available starting materials have been depleted, the assembler consumes itself, resulting in self-destruction of the aggregates. Whereas this system in no way guides the polymerization reaction by positioning reactive molecules with atomic precision, as envisioned by Drexler, it functions by one of the reactants creating an encapsulated self-assembled space. Like Drexlers vision of an assembler, this confined space brings the reactants together, allowing for a reaction to take place. This system provides opportunities to design transient supramolecular systems capable of performing functions such as controlled uptake of a systems component and synthesis as well as polymerization.

## Results

### Lifespan of the assembler

Experiments with the assembler were performed for various temperatures, pH, reactant concentrations, and stirring rates. In all cases, the assembler’s lifespan is characterized by the same three distinct phases; formation, function, and self-destruction. The transition between these three phases is fluid, showing no abrupt changes.

### Formation of the assembler

**Fig. 2:**
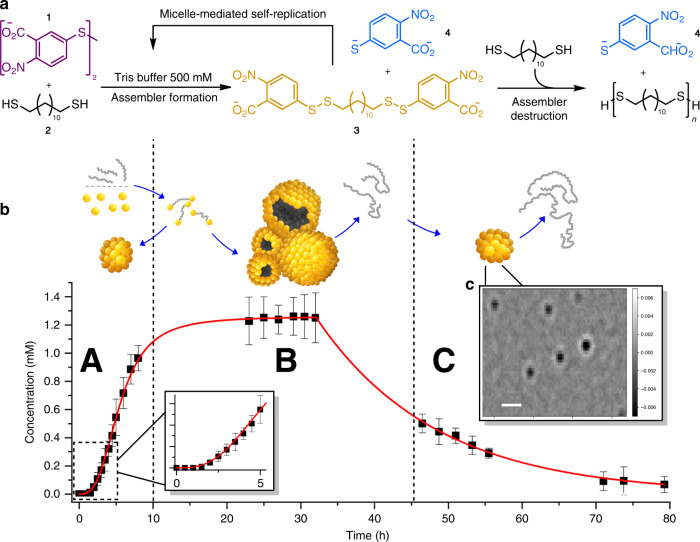
Evolution of the concentration of molecular assembler building block over time. **a**, **b** The reaction between phase-separated disulfide (**1**) and thiol (**2**) results in formation of (bola)amphiphilic compound **3** that self-assembles into supramolecular micelles (A). These micelles aid in solubilizing the apolar thiol into the aqueous layer, thereby creating an autocatalytic feedback (see insert). The assembler can also use the dissolved thiol in a second type of reaction, thereby producing disulfide polymers. Although this reaction results in the destruction of its building block (compound **3**), continued production balances that reaction, resulting in a constant concentration of amphiphile (B). When the supply of thiol (**2**) gets depleted, amphiphile destruction outcompetes formation, resulting in a decrease in concentration (C). Polymers continue to grow during this period. **c** iSCAT image of the molecular assembler, composed of (bola)amphiphilic compound **3** show spherical particles (scale bar: 1 µm). The average mass of these particles was calculated at 200 kDa. Note that this image shows the ratiometric contrast of particles, rather than their actual size. Error bars represent the standard deviation. Source data are provided as a Source Data file. A range of reaction conditions were tested, all yielding a similar concentration profile to this example (50 mM **1**, pH 8.0, 40 °C, 200 rpm, concentrations were determined using UPLC).

Our molecular assembler forms from hydrophilic disulfide **1** in buffered water and hydrophobic dodecane-1,12-dithiol (compound **2**). The reaction between the two reagents results in the formation of (bola)amphiphilic molecule **3**. These amphiphiles self-assemble into supramolecular aggregates^26,28,29^, which constitutes the molecular assembler (Fig. 1a). Analysis of these aggregates using interferometric scattering microscopy (iSCAT)^30^ showed aggregation in the form of micelles, with an average mass of ~200 kDa (corresponding to an aggregation number of 320, Fig. 1, bottom right). Dynamic light scattering (DLS) measurements further indicated aggregation in the form of micelles, with particle sizes ~7 nm. These micelles serve as a catalyst for their own formation, by solubilizing compound **2**, thereby increasing the contact area between the two reactants. Hence, this is an example of physical autocatalysis^25,31–34^. As more amphiphile is formed, the dissolution rate of the apolar reactant (**2**) is increased. This in turn, results in exponential growth of the concentration of **3**, which is characteristic for autocatalytic reactions (Fig. 2).

### Functional molecular assembler

In contrast to most autocatalytic reactions where product concentration increases until the starting materials are consumed^26,35^, here the amphiphile concentration reaches a steady state. Although the assembler continues to produce its amphiphilic building blocks, the solubilized dithiol also reacts with this building block in a second reaction. The amphiphile can react with two equivalents of thiol to produce a (linear) trimer of disulfide. This process results in destruction of the amphiphilic building block. As this disulfide trimer, produced in the destruction step, still contains two thiol moieties, it can react with additional amphiphile to result in further chain growth. The driving force of the overall process is the conversion of high-energy disulfide **1** and thiol **2** to the thermodynamically favored disulfide polymer and thiol **4**. In previous examples of autocatalytic reactions on monofunctional reactants, the formation of the bisalkyl-disulfide as the thermodynamic product marks the end of reactivity. In the case of compound **2**, the presence of functional thiol groups on both ends of the alkyl chain that remain intact upon disulfide formation, constitutes a continuous driving force for further polymer growth. The combined processes during this phase can be regarded as a metabolic-cycle-like system, in which the assembler acts as the catalyst (Fig. 1b). It produces and consumes its own building block at an equal rate, thereby forming the disulfide polymers. The equal rates of formation and destruction of amphiphile imply that its concentration is constant over time. This steady state with respect to the amphiphile concentration is maintained as long as the supply of reactants (compounds **1** and **2**) is sufficient.

### Self-destruction of the assembler

**Fig. 3:**
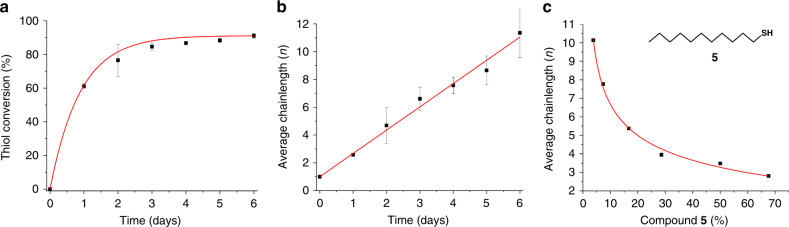
Growth of disulfide polymers. **a** The molecular assembler drives the conversion of thiol (**2**) into disulfide polymers. Numbers were obtained using NMR analysis of the produced polymers and were further confirmed using gel permeation chromatography (GPC). **b** Based on the conversion of thiol to disulfide, the average chainlength of the produced polymers can be calculated. **c** Average polymer length after 6 days of polymerization in the presence of 1-dodecanethiol (**5**). The amount of 1-dodecanethiol (which acts by capping the growing polymers, stopping further chain elongation) as percentage of the total thiol amount is given on the *x* axis. Error bars represent the standard deviation. Source data are provided as a Source Data file.

In our experiments, an excess of compound **1** is used, implying that the lifetime of the assembler is solely limited by the availability of **2**. As the supply of compound **2** becomes smaller, the rate of formation of the amphiphile building block is reduced. As amphiphile destruction does not necessarily require **2**, but can also use previously formed polydisulfides (which still contain thiol moieties), this process is hampered to a lesser extent (Fig. 1c, note that although amphiphiles containing multiple alkyl linkers could in principle also be formed, they were only present in significantly lower concentrations). As amphiphile destruction now dominates formation, its concentration diminishes over time. Self-destruction of the assembler is not an abrupt process, but happens gradually, whereas the amount of available **2** decreases. During this period, the assembler keeps consuming its own building blocks, resulting in continuous polymer growth. The diminishing amount of amphiphile does result in a slower rate of polymerization over time (Fig. 3a). When eventually all thiol **2** has been depleted, consumption of the final building blocks results in (self)-destruction of the assembler, making it an example of a transient species.

### Polymer production

During these experiments, the growing disulfide polymers precipitate from the solution and can be easily collected by filtration. Polydisulfides are a useful class of compounds because the disulfide bond is easily cleaved to cause polymer destruction when desired^36,37^. This property has been exploited by others in the use of cell-penetrating poly(disulfide)s (CPD)^38^, which can be used to deliver drugs, proteins, and other compounds across cell-barriers into cells^39,40^. As the final destination of CPDs is highly dependent on polymer length, it is vital to exert control over polymer length^40^.

For our system, the polymer size can be selected by filtrating the reaction mixture at the right time, as the average polymer length increases with time and temperature (Fig. 3b and SI). Alternatively, to exert a higher degree of control over the average polymer length 1-dodecanethiol (**5**) can be added to induce polymer capping^41^. Once **5** has been incorporated on both sides of a polymer molecule, no more thiols are present, stopping further polymerization. The ratio of monothiol (**5**) to dithiol (**2**) thus determines the final average chainlength (Fig. 3c). This also implies that the resultant polymer is devoid of any reactive thiol groups. The polymerization process was followed over time in detail using both nuclear magnetic resonance (NMR) and gel permeation chromatography (GPC) (see ESI). Depolymerization of the final products could be achieved by treatment with dithiothreitol, thereby regenerating dithiol **2**.

### Influence of temperature and pH

**Fig. 4:**
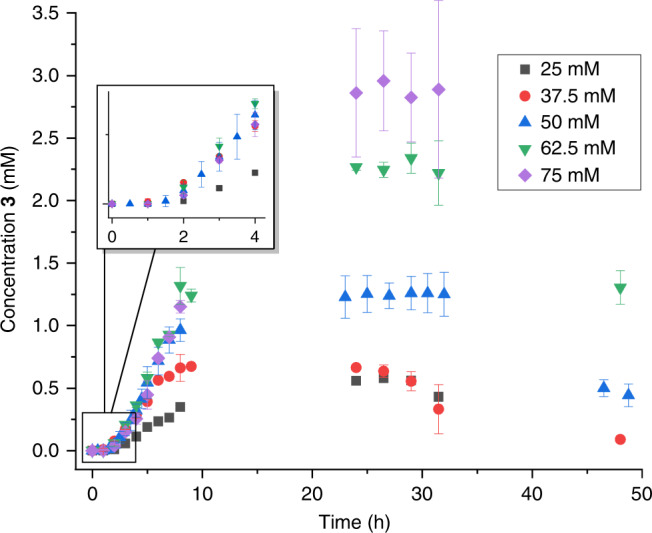
Effect of the initial concentration of 1 on the assembler concentration. Increasing the initial concentration of compound **1** results in faster formation of **3**, whereas the destruction rate is unchanged, increasing the concentration of **3** throughout the experiment. All experiments show an initial period of autocatalytic growth (see insert), followed by a period of constant [**3**] and finally destruction of the assembler. Concentrations were determined using UPLC, whereas error bars represent the standard deviation. Source data are provided as a Source Data file.

Although tuning the reaction time and/or using polymer capping provide adequate control over the average polymer size, the course of the experiments proved sensitive to several reaction parameters. Variation of the reaction temperature simply resulted in changes in polymerization speed and lifetime of the assembler (see SI). Tuning the reactant concentration ([**1**]) resulted in much larger variations, involving significant changes in the concentration of the assembler’s building blocks ([**3**]) throughout the experiment (see Fig. 4). This can be rationalized by a scenario where increasing [**1**] results in faster formation of surfactant **3**, whereas destruction of **3** (due to polymerization) is independent of [**1**], resulting in an overall increase in [**3**]. In addition the effect of pH on [**3**] was investigated. By increasing the pH, more negatively charged alkylthiol becomes present, potentially resulting in both faster formation and destruction of **3**. The lifetime of the system is significantly shorter when pH is increased (see supplemental fig. S3.1). In addition, the maximum concentration of **3** in these experiments significantly decreased, indicating that destruction of **3** is more sensitive to pH than formation. Finally, we investigated the effect of elongating the chainlength of the dithiol (from 12 to 20 carbons) on the polymerization process. Although the corresponding surfactant aggregated in the form of vesicles instead of micelles, these vesicles played no role in the (very slow) polymerization process (see SI).

## Discussion

Although the exact molecular assembler envisioned by Eric Drexler will remain elusive to chemists, man-made analogs that approximate the concept have been recently devised^18,42^. In this work, we have developed a supramolecular system that adheres to several key requirements of Drexler’s molecular assembler. The primitive molecular assembler described in this work, self-aggregates via the autocatalytic reaction of two phase-separated components, and reaches a steady state where a reactant is consumed and transformed into polymers. The initial period of autocatalytic growth adheres to the requirement of a molecular assembler to display early-state self-replication. Although Drexler proposed that this self-replication should be followed by reprogramming of the assembler to allow for the production of the required product, in the case of our assembler, this process occurs spontaneously. The assembler starts balancing the continuous generation of its building blocks with a second (destructive) reaction, resulting in the production of the intended disulfide polymers. Although the assembler exists as a transient species, it can maintain its concentration at a constant level for extended periods of time. The consumption of bis-sulfide fuel keeps the system in this out-of-equilibrium state so long as reactant is available. It thereby appears able to maintain its concentration through a primitive form of dynamic kinetic stability. The sensitivity of the system to changes in [**1**] and pH, point toward the system being controlled by kinetic rather than thermodynamic factors. After one of the reagents, required for the assembler’s building block formation, gets depleted the assembler will continue to produce polymers, resulting in self-destruction of the system. Overall, this study provides an alternative direction for the design of functional systems based on balancing the incorporation, and consumption of the assembler’s building blocks in a series of steps, at least one of which must be irreversible (under the operating conditions) to provide the sense of directionality or asymmetry necessary to allow the assembler to operate in time. Although this system in no way allows the production of products by positioning reactive molecules with atomic precision, it relies on an encapsulated self-assembled space to direct the reaction between otherwise (phase)-separated reactants. This solves the common challenge in the design of molecular assemblers of realizing nanoproduction line-like processes, where overcoming diffusion and Brownian motion is difficult^26,43,44^. The concept of using encapsulation to create confined reaction environments is by itself not new. Confinement can result in altered chemical properties, which in turn can change reaction rates and selectivity^45^. Encapsulation has been extensively used on enzymes, using surfaces^46^ as well as three-dimensional compartments such as (giant) vesicles, protein cages or (reverse) micelles^47^. This approach is not limited to a single enzyme, but can involve a series of catalytically active species, creating a cascade of chemical reactions^48^. In contrast to our current work, the supramolecular structures providing the confinement in these examples are generally composed of a secondary compound, and not the (catalytically) active species itself. Although our present system relies on supramolecular structures that spontaneously emerge and are controlled by reaction kinetics, other strategies which rely on external stimuli (light, chemical, etc.) can be envisaged that would provide spatiotemporal control over catalytically active aggregates.

In summary, a spontaneously emerging molecular assembler, able to produce disulfide polymers was discovered. Two phase-separated components react to form amphiphiles that self-aggregate into the assembler, in the form of supramolecular micelles. An initial period of exponential growth owing to self-replication is followed by a period of constant amphiphile concentration, which is sensitive toward experimental conditions, and heavily dependent on pH and reactant concentration. During this period, the assembler’s building blocks are both produced and consumed, whereas polymers are produced. By adjusting temperature and reaction time, or by adding a reagent that causes polymer capping, control over polymer size can be achieved. Once the starting material has been depleted, the assembler continues to use its own building blocks to allow further polymerization, resulting in self-destruction of the system. Throughout this process, the assembler uses its self-encapsulated space to bring the reactants for the polymerization process into contact. Although the reactants are not placed together with atomic precision (as for Drexler’s assembler), the confined space created by the self-assembly of one of the reactants drastically increases their contact. This effectively overcomes the necessity of diffusion and Brownian motion that would otherwise limit the feasibility of molecular assembly-line processes. This work thereby provides alternative directions for the development of functional supramolecular systems.

## Methods

### General experimental details

Reagents were obtained from Sigma-Aldrich (except for **1**, which was obtained from Fluorochem) and were used without further purification. Synthesis of compounds **2** and **3** is described in the Supplemental information. Flash column chromatography was performed using silica gel (60 Å, 0.033–0.070 mm, BDH). Thin layer chromatography analyses were performed on Merck Kiesegel 60 F254 0.25 mm precoated silica plates. All ^1^H NMR and ^13^C NMR spectra were recorded using a Bruker AVIII HD Nanobay 400 MHz spectrometer. Chemical shifts are given in ppm with respect to tetramethylsilane as an internal standard (Supplementary Figs. 20–23). High-resolution mass spectra were obtained with a Bruker MicroTOF apparatus using electrospray ionization.

### Polymerization experiments

For the polymerization experiments, a total of 80 mg compound **1** (0.2 mmol), 4 mL, 0.5 M TRIS buffer (pH 8.00) and a small stirring magnet (0.5 cm) were added to a 7 mL flat vial. The solution was stirred at 200 rpm and the vial was heated to 40 °C. After compound **1** had completely dissolved, 27 µl compound **2** (23 mg, 0.09 mmol, m.p. = 34 °C) was carefully added on top of the water layer as an oil (by heating it to 40 °C), and stirring was continued. Samples were taking regularly, by carefully extracting 20 µL of the aqeous layer. The extracted solution was immediately added to 1 mL of a 62 mM aqeous solution of maleimide (containing 0.16 mM 3-methyl-2-nitrobenzoic acid as an internal reference) to quench all remaining thiols. Samples were analyzed as described in the ultra performance liquid chromatography (UPLC) section. To determine the growth of the disulfide polymers (present as a light yellow solid that floated on top of the water layer), some solid was carefully extracted using a spatula and subsequently dried using paper tissue. The average chainlength was determined based on the thiol to disulfide ratio, that could be accurately determined using ^1^NMR spectroscopy and GPC analysis (Supplementary Figs. 1–4, 23).

### GPC analysis

GPC analysis of polydisulfides was performed on a Shimadzu LC-20AD instrument, equipped with a refractive index (RI) detector and two PSS SDV 5 μm linear M columns. High-performance liquid chromatography grade tetrahydrofuran was used as the eluent at 1.0 mL/min at 30 °C. Samples were passed through 0.2 μm olytetrafluoroethylene filters prior to analysis. Monodisperse polystyrene standards were used for calibration. Number average molar mass (*M*_n_), weight average molar mass (*M*_w_), and dispersity (*Ɖ*) were calculated using Shimadzu LabSolutions GPC analysis program. Pure compound **2** (monomer) and three samples obtained at consecutive time points in the polymerization experiment were analyzed using both ^1^H NMR and GPC. In addition, a polymer sample of the final time point was depolymerized (see Supplemental information) and subsequently analyzed using NMR and GPC to show conversion back to the monomer. Comparison between the two methods, as well as the unmodified GPC data, are given in Supplementary Table 1. In all cases, good agreement between both analysis methods was obtained.

### UPLC analysis

The concentrations of compounds **1**, **3**, and **4** were determined using a Waters Acquity ultra performance liquid chromatography UPLC H-Class system with photodiode array detector. All data were processed using Empower software. An Acquity UPLC BEH C18 column (130 Å, 1.7 μm, 2.1 mm × 50 mm) was used. A mixture of water: MeCN: 5% TFA in H2O with a gradient of 93:2:5 changing to 0:95:5 over 5 min was used as the mobile phase. All peaks were analyzed at a wavelength of 330 nm (Supplementary Fig. 19).

### DLS analysis

Aggregate sizes were analyzed with DLS measurements, using a Malvern Zetasizer Nano ZEN5600 apparatus. All samples were prepared by dissolving **3** in a 0.5 M TRIS buffer (pH 8.00) and were filtered using a microfilter (poresize 0.22 µm) before measurements. Samples were placed in a 1 mL plastic disposable cuvette and were heated to 60 °C using an equilibrated heating probe. Measurements on solution containing compound **3** gave a maximum intensity for a particle size of ~7 nm, regardless of the concentration, which sizes were further confirmed by iSCAT measurements (Supplemental Figs. 6–18).

### Determination of the critical micelle concentration

Ring tensiometry measurements were used to determine the critical micelle concentration (CMC) of compound **3**. Samples with varying concentrations of **3** in a 0.5 M TRIS buffer (pH 8.00) were prepared and their surface tension was determined at 25 °C (in triplicate) using a Kruss K10ST tensiometer. The CMC (0.011 mM) is the point at which an increase in the concentration of **3** no longer results in a decrease in the surface tension (Supplemental Fig. 5).

## Supplementary information

Supplementary Information

## Data Availability

Correspondence and requests for materials should be addressed to S.P.F. The source data underlying Figs. 2, 3a–c, and 4 are provided as a Source Data file. Source data are provided with this paper.

## Source data

Source Data
