# Supplementary material for: A molecular assembler that produces polymers
Source: Nat Commun. 2020 Aug 19;11:4156. doi: 10.1038/s41467-020-17814-0 (PMC7438324; doi:10.1038/s41467-020-17814-0)
Supplement: Supplementary file 1 — Supplementary Information [file 41467_2020_17814_MOESM1_ESM.pdf]

Supplementary Information

A Molecular Assembler that Produces Polymers

Engwerda et al.

A Molecular Assembler that Produces Polymers

Anthonius H. J. Engwerda & Stephen P. Fletcher

Supplemental Information

Supplementary methods 3

Polymerization experiments 3

Seeding experiments 5

pH depedent experiments 6

Ring tensiometry data 7

GPC measurements 8

DLS measurements 10

iSCAT measurements 13

Effect of the chainlength of the alkylthiol 16

Synthesis of compounds 21

UPLC calibration 22

Supplementary discussion 23

Polymerization mechanism 23

Supplementary figures 24

NMR spectra 24

Supplementary References 28

Supplementary methods

Polymerization experiments

*This section describes the conditions used in experiments involving the molecular assembler. The standard conditions used for figure 1 are described in the general experiment. For the experiments of figure 4, the same procedure was used, only changing the amount of compound **1** at the start of the experiment. Concentrations of compound **3** were determined by sampling the reaction mixture (see Sampling of the solution) and consequent analysis of these samples using UPLC. Experiments studying the growth of polymers (figure 3) were carried out in an identical way, now sampling the polymers instead of the aqeous layer (see Sampling and analysis of the polymer product). Polymer were analysed using both GPC and NMR.*

General experiment:

In a typical experiment, a total of 80 mg compound **1** (0.2 mmol), 4 mL, 0.5M TRIS buffer (pH 8.00) and a small stirring magnet (0.5 cm) were added to a 7mL flat vial. The solutions were stirred at 200 rpm (unless stated otherwise) and the vial was heated to the required temperature (usually 40 ˚C). After compound **1** had completely dissolved, 27 µl compound **2** (23 mg, 0.09 mmol) was carefully added on top of the water layer as an oil (by heating it to ~40˚C), and stirring was continued.

Sampling of the solution:

Samples were taking regularly, by carefully extracting 20 µL of the aqeous layer. The extracted solution was immediately added to 1 mL of a 62 mM aqeous solution of maleimide (containing 0.16 mM 3-methyl-2-nitrobenzoic acid as an internal reference) to quench all remaining thiols. Samples were analyzed as described in the UPLC section.

Sampling and analysis of the polymer product:

Formed polymers were present as a light yellow solid that floated on top of the water layer upon precipitation. Some solid was carefully extracted using a spatula and subsequently dried using paper tissue. The average chainlength was determined based on the thiol to disulfide ratio, that could be accurately determined using ^{1}NMR spectroscopy (see NMR section and Supplementary figure 1).

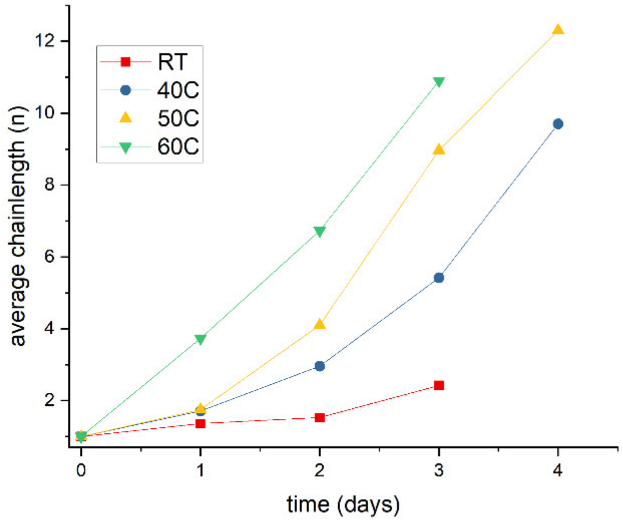

Supplementary figure 1 Effect of the temperature on polymer growth. Evolution of the average chain length of disulfide polymers, formed from the biphasic reaction between compounds **1** (25 mM at pH 8) and **2** at various temperatures.

Additional analysis of the polymer chainlength using mass spectrometry (using either ESI with QM detection, ESI with TOF detection or MALDI) proved unsuccesful. Further analysis and confirmation of NMR data was therefore achieved using gel permeation chromatography (GPC), using polystyrene as a reference.

Depolymerization:

A total of 17 mg polymer and 88 mg racemic dithiothreitol (8 eq) was dissolved in 4 mL THF. The clear solution was stirred for 72 hours, after which the solvent was reduced. The mixture was run over a short silica column (100% hexanes) to yield the depolymerized product (1,12-dodecanedithiol) as a colourless solid (15 mg, 88%). The resultant monomer was analyzed using both NMR and GPC.

Seeding experiments

Experiments were performed as described in section 1, but now the reaction was seeded with compound **3** before addition of thiol **2**. Addition of small amounts of **3** (<0.5 mM) resulted in elimination of the lag period, while moving towards the same equilibrium concentration. Addition of higher concentrations of **3** resulted in a decrease of **3**, although a period of constant [**3**] was never observed (see supplementary figure 1). The autocatalytic nature of the reaction was further established by following the initial rate of formation of compound **4** as a function of seed concentration (Supplementary figure 2)

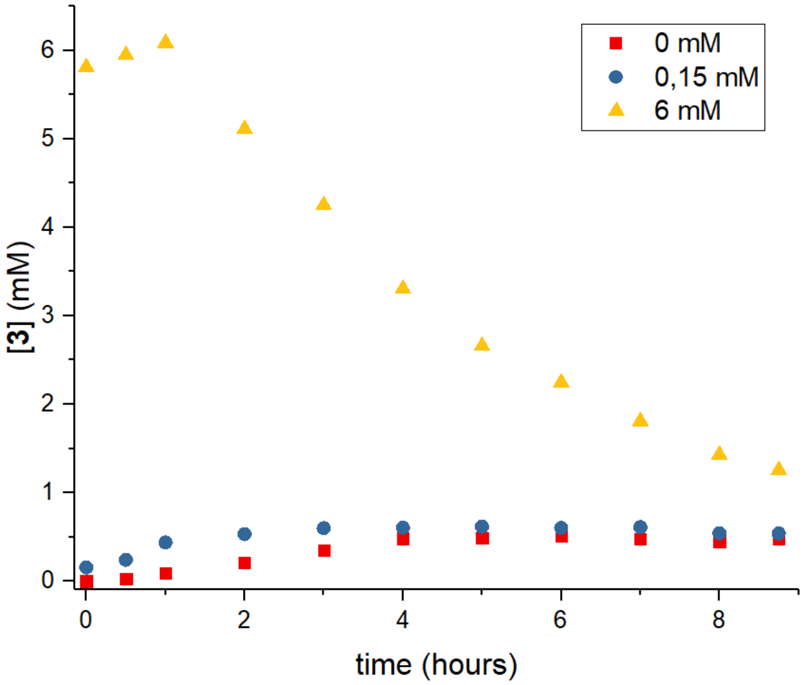

Supplementary figure 2 Effect of seeding on the concentration of 3. Polymerization experiments monitoring the concentration of compound **3** starting with varying amounts of seeds. Experiments were performed with 25 mM **1** and at 40˚C.

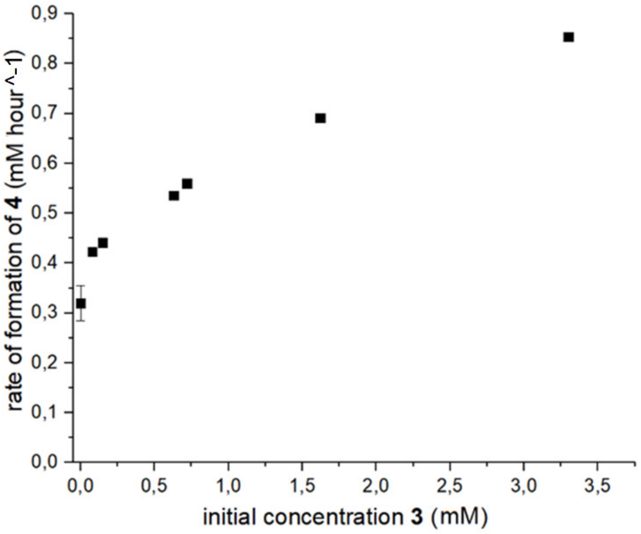

Supplementary figure 3 Effect of seeding on the initial rate of formation of 3. Initial rate of formation of compound **4** a function of the seed concentration of **3**. The observed positive correlation further supports the autocatalytic nature of the reaction,

pH depedent experiments

We aimed to determine how the molecular assembler responds to changes in its environment, specifically how the concentration of **3** is influenced by various reaction conditions. Whereas the temperature simply sped up the lifecycle of [**3**] (but did not affect its concentration profile), the initial concentration of **1** is a key parameter (see Fig 4 of main manuscript). In addition the effect of pH on [**3**] was investigated (Supplementary figure 4).

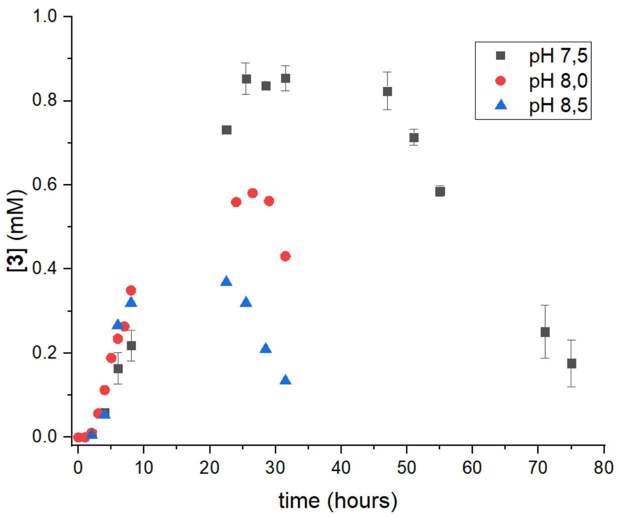

Supplementary figure 4 Effect of the pH on the assembler's lifetime. Unseeded reactions following the formation of surfactant **3**, starting from various pH values using 25mM **1**. Error bars represent the standard deviation.

Ring tensiometry measurements

Ring tensiometry measurements were used to calculate the critical micelle concentration (CMC) of compound **3**. Surface tension is plotted against ln[**3**], whereby the point from which the surface tension no longer decreases corresponds with the CMC. This can be calculated using the intercept of the two drawn lines (Supplementary figure 5):

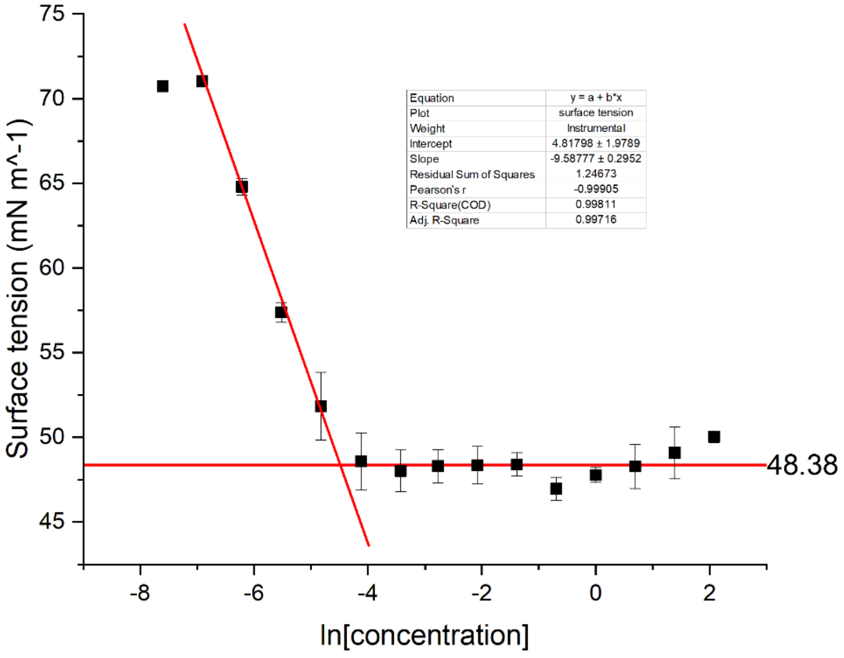

Supplementary figure 5 Surface tension dependency on [3]. Plot of the surface tension versus [**3**] in a TRIS buffer (pH 8.0). The intercept of both lines corresponds to the CMC. Error bars represent the standard deviation

$$\text{Surface tension} = 4.82 - 9.59 * \ln(\text{concentration})$$

$$\text{Surface tension} = 48.38$$

$$4.82 - 9.59 * \ln(\text{concentration}) = 48.38$$

$$\underline{\text{Critical Micelle Concentration (CMC)} = 0.011\ \text{mM}}$$

GPC measurements

GPC analysis of polydisulfides was performed on a Shimadzu LC-20AD instrument, equipped with a Refractive Index (RI) detector and two PSS SDV 5 µm linear M columns. HPLC grade THF was used as the eluent at 1.0 mL/min at 30 °C. Samples were passed through 0.2 µm PTFE filters prior to analysis. Monodisperse polystyrene standards were used for calibration. Number average molar mass (M_n), weight average molar mass (M_w), and dispersity ($Đ$) were calculated using Shimadzu LabSolutions GPC analysis program. Pure compound **2** (monomer) and three samples obtained at consecutive time points in the polymerization experiment were analysed using both ^{1}H NMR and GPC. In addition, a polymer sample of the final time point was depolymerized and subsequently analyzed using NMR and GPC to show conversion to the monomer. Comparison between the two methods, as well as the unmodified GPC data, are given in supplementary table 1. In all cases, good agreement between both analysis methods was obtained.

Supplementary table 1 Analysis of polymer samples using GPC and NMR.

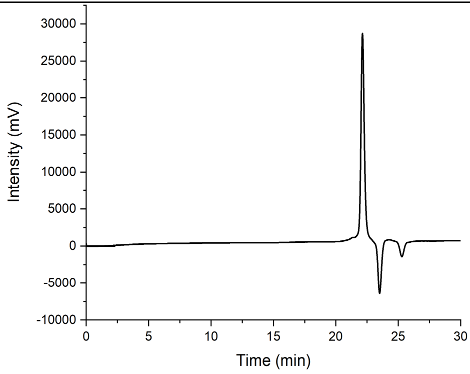

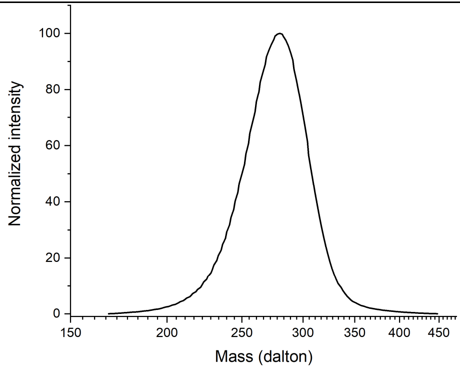

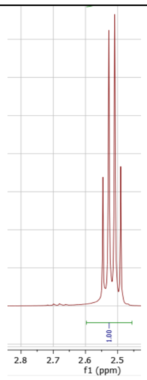

Average chainlength = 1 (compound **1**) **Mn (GPC) = 274** (D=1.01) **Mn(NMR)=234**

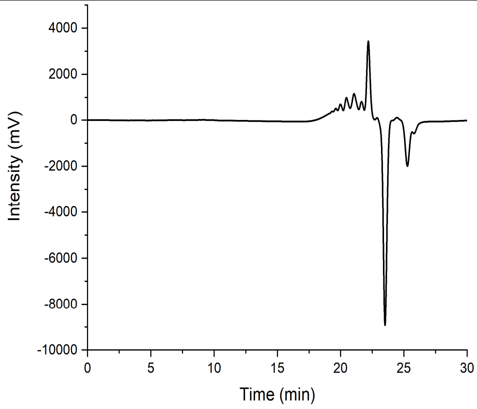

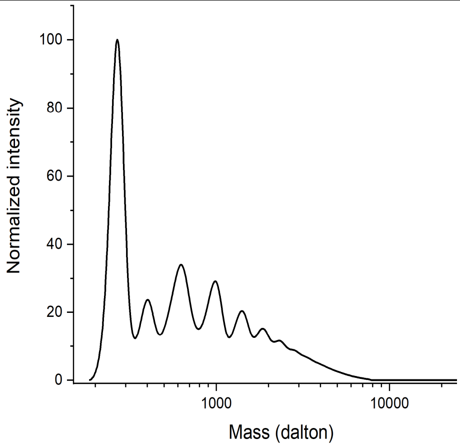

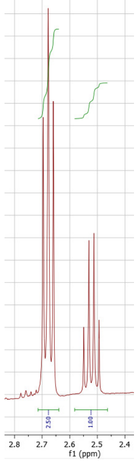

Average chainlength = 2.47 **Mn(GPC)=465** (D=1.95) **Mn(NMR)=812**

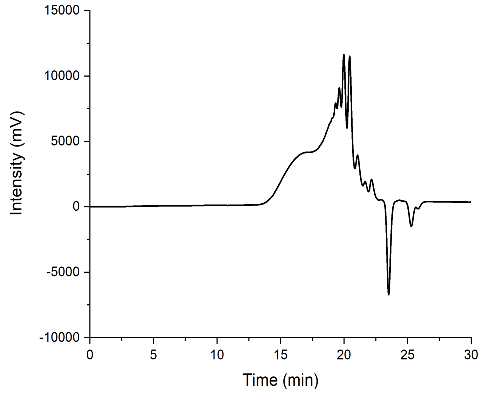

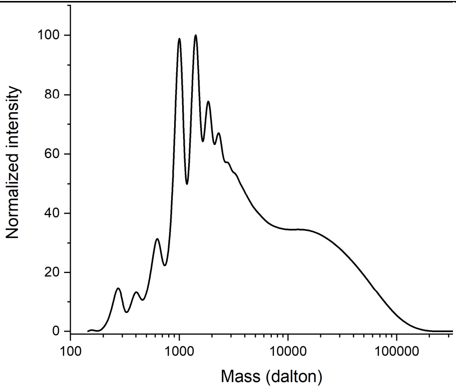

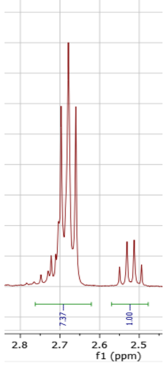

Average chainlength = 8.37 **Mn(GPC)=1802** (D=5.74) **Mn(NMR)=1959**

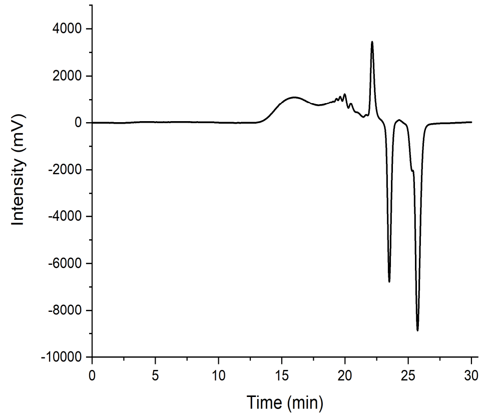

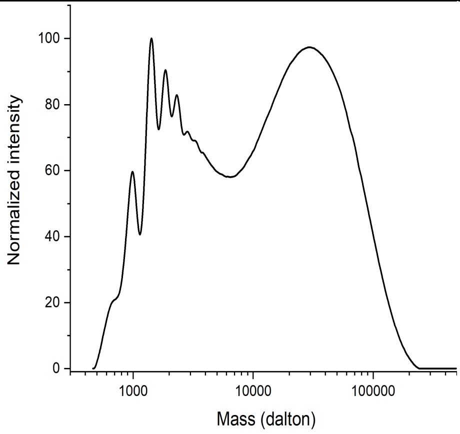

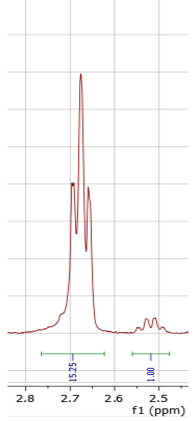

Average chainlength = 16.25 **Mn(GPC)=4054** (D=5.99) **Mn(NMR)=3803**

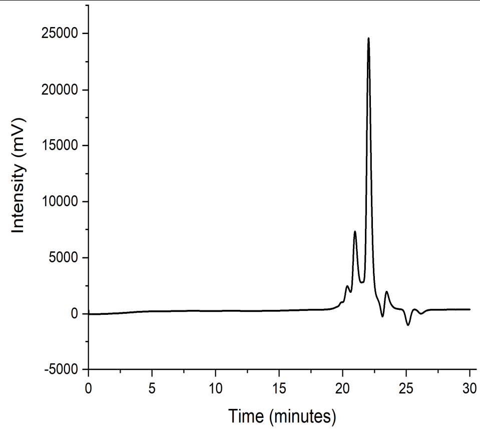

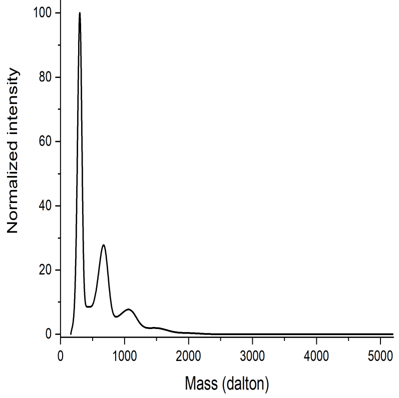

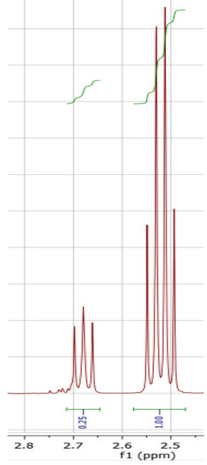

Average chainlength = 1.25 (depolymerized material) **Mn(GPC)=350** (D=1.23) **Mn(NMR)=290**

Dynamic Light Scattering measurements

Analyses were performed using a Malvern Zetasizer Nano ZEN5600 model system recording particle and molecule size. Instrument control and data processing were performed using Zetasizer software. Disposable plastic cuvettes were used with 1.0 mL of sample solution. Measurements were repeated thrice for every concentration. Measurements were done using an equilibrated heating probe at 60 °C, setting the appropriate parameters for water. All samples were prepared in a 0.5 mM TRIS buffer (pH 8.00) and were filtered using a microfilter (poresize 0.22 µm) prior to measurement.

Measurements on solution containing compound **3** gave a maximum intensity for a particle size of around 7 nm at Ph 8.0, regardless of the concentration. The aggregate size did depend on the pH, by showing a trend of decreasing size for increasing pH (Supplementary figures 6-9):

pH:	Average aggregate size:	Standard deviation (of maximum intensity):
7.5	8.26	0.34
8.0	6.86	0.38
8.5	6.88	0.32
9.0	6.22	0.11

			Size (d.n...	% Intensity:	St Dev (d.n...
Z-Average (d.nm):	5.841	Peak 1:	6.349	90.1	2.680
PdI:	0.254	Peak 2:	608.8	9.9	348.5
Intercept:	0.831	Peak 3:	0.000	0.0	0.000
Result quality	Good				

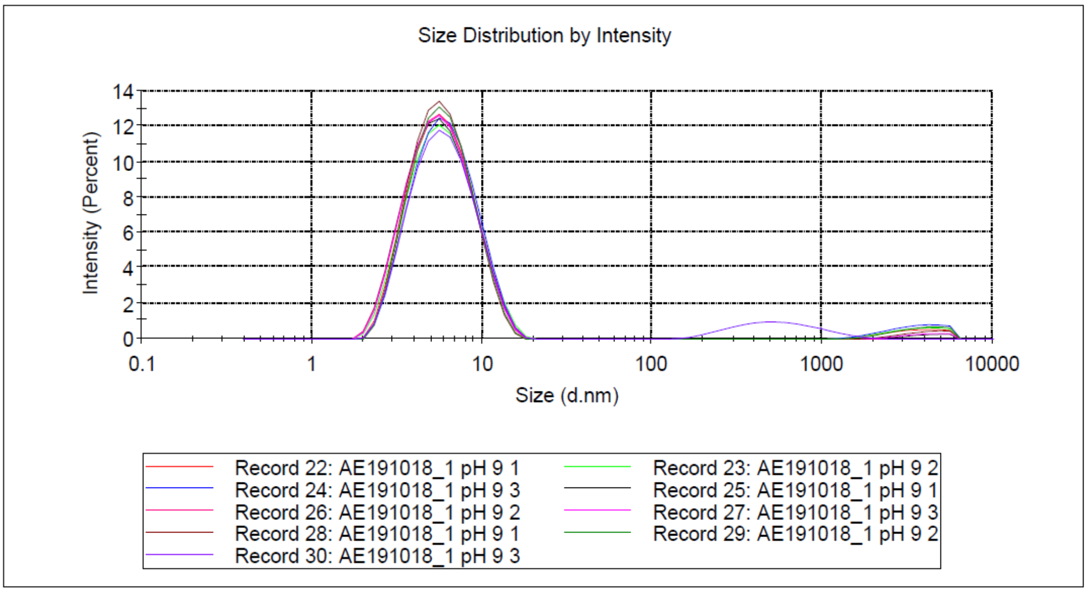

Supplementary figure 6 DLS measurement on a 4 mg/mL (6.4 mM) solution of compound 3 at pH 9.0.

			Size (d.n...	% Intensity:	St Dev (d.n...
Z-Average (d.nm):	5.464	Peak 1:	6.659	96.3	2.964
PdI:	0.239	Peak 2:	3649	3.7	1195
Intercept:	0.826	Peak 3:	0.000	0.0	0.000
Result quality	Good				

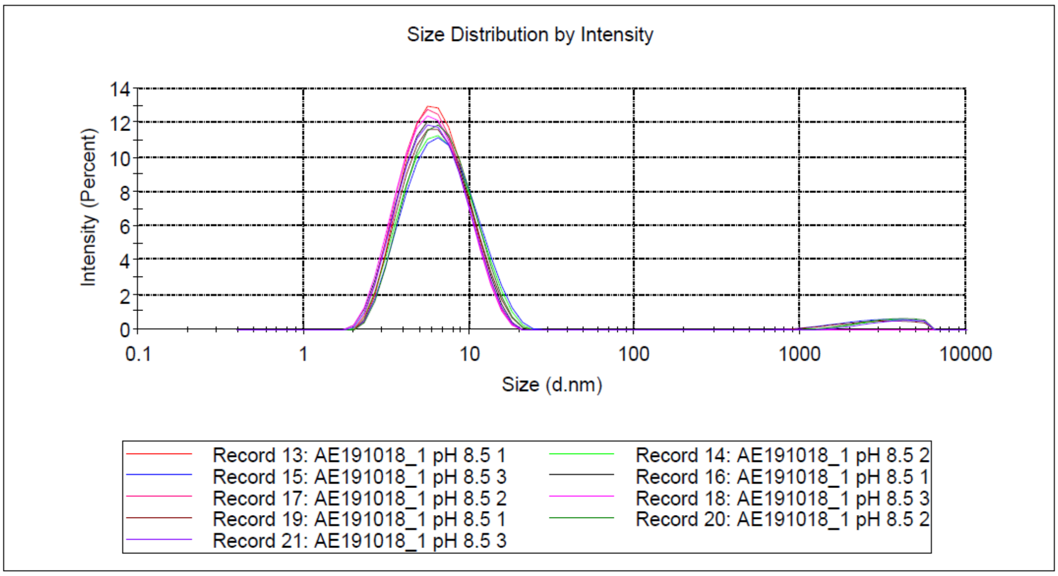

Supplementary figure 7 DLS measurement on a 4 mg/mL (6.4 mM) solution of compound 3 at pH 8.5.

			Size (d.n...	% Intensity:	St Dev (d.n...
Z-Average (d.nm):	5.324	Peak 1:	6.329	100.0	2.217
PdI:	0.152	Peak 2:	0.000	0.0	0.000
Intercept:	0.850	Peak 3:	0.000	0.0	0.000
Result quality	Good				

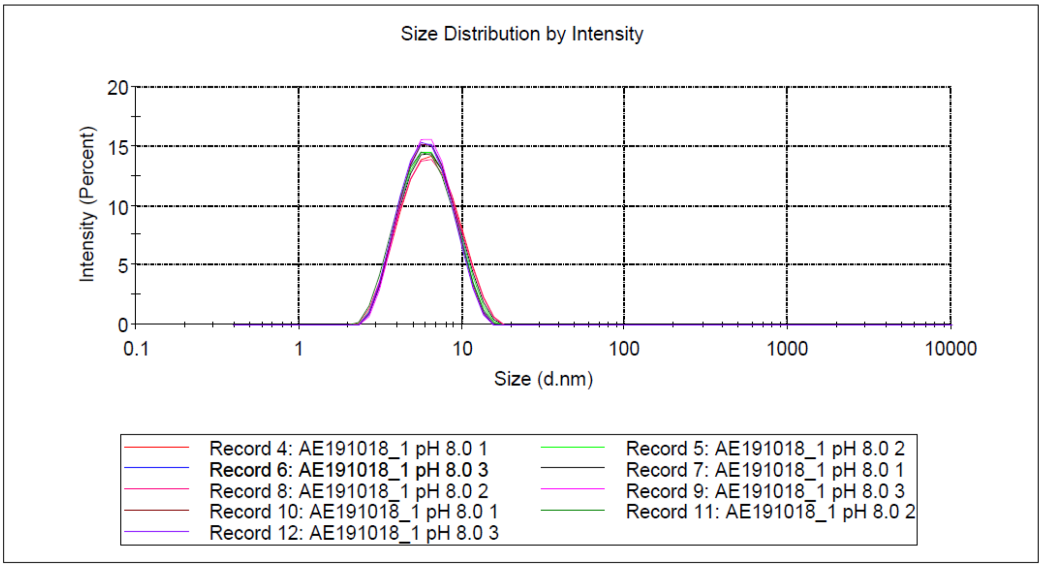

Supplementary figure 8 DLS measurement on a 4 mg/mL (6.4 mM) solution of compound 3 at pH 8.0.

			Size (d.n...	% Intensity:	St Dev (d.n...
**Z-Average (d.nm):**	6.863	**Peak 1:**	7.933	100.0	2.889
**PdI:**	0.127	**Peak 2:**	0.000	0.0	0.000
**Intercept:**	0.910	**Peak 3:**	0.000	0.0	0.000
**Result quality**	**Good**				

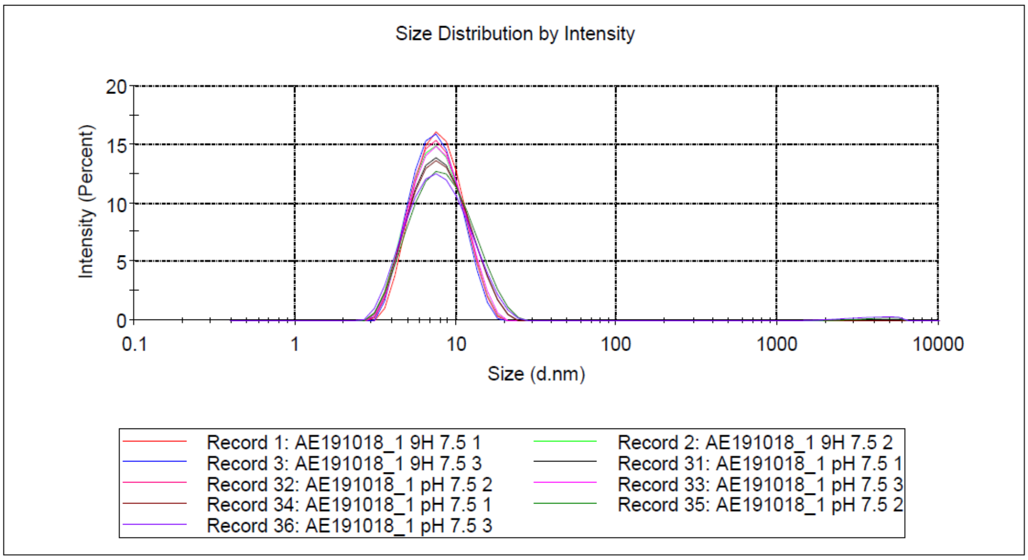

Supplementary figure 9 DLS measurement on a 4 mg/mL (6.4 mM) solution of compound 3 at pH 7.5.

Interferometric scattering microscopy (iSCAT) measurements

The iSCAT experimental set-up is similar to that described by Young et al,[1] with a 532 nm diode laser used as the incident light source. Frames were recorded at 1 kHz with an exposure time of 0.98 ms, using a CMOS camera. Focus in the z axis is maintained using an autofocus system relying on the total internal reflection (TIRF) of a 638 nm beam. Instrument control was performed using the custom software written in LabView.

Data processing. Data processing was performed using the custom software written in Python, as described elsewhere.[1] In brief, differential imaging was achieved by subtracting sets of images temporally offset by a time Δt. The signal-to-noise ratio was then improved by spatially (3 x 3 binning) and temporally averaging the differential images (50 images).

Particle detection was performed as described by Young et al.[16] Briefly, diffraction-limited spots were identified by the software, and fitted to the 2D Gaussian function to give the ratiometric contrast value.

Coverslips and sample preparation for iSCAT analysis. Samples for iSCAT analysis were prepared in a TRIS buffer (pH 8), using a 0.004 mM concentration of compound **3**.

Glass coverslips (no. 1.5, 24 x 50 mm, VWR; and 24 x 24 mm, VWR) were cleaned by sequential sonication in MilliQ water, isopropanol and MilliQ water (5 min each), and dried under stream of nitrogen. Chambers were prepared by attaching a (24 x 24) coverslip on top of a (24 x 50 mm) one with double-sided tape. All coverslips and chambers were prepared on the day of the analysis or the day before.

Contrast-to-mass (C2M) calibration was performed in the corresponding buffer solution, since the C2M conversion may change slightly as a result of buffer content. The calibration protocol included measurement of a protein oligomer solution, with masses of 90, 180, 360 and 540 kDa. Each calibration experiment was analysed using same software as described above. The mean peak contrast was determined in the software using Gaussian fitting. The mean contrast values from the calibration protein solution were then plotted (Supplementary figure 10) and fitted to a line, y=bx, with y–contrast , x–mass and b–C2M calibration factor.

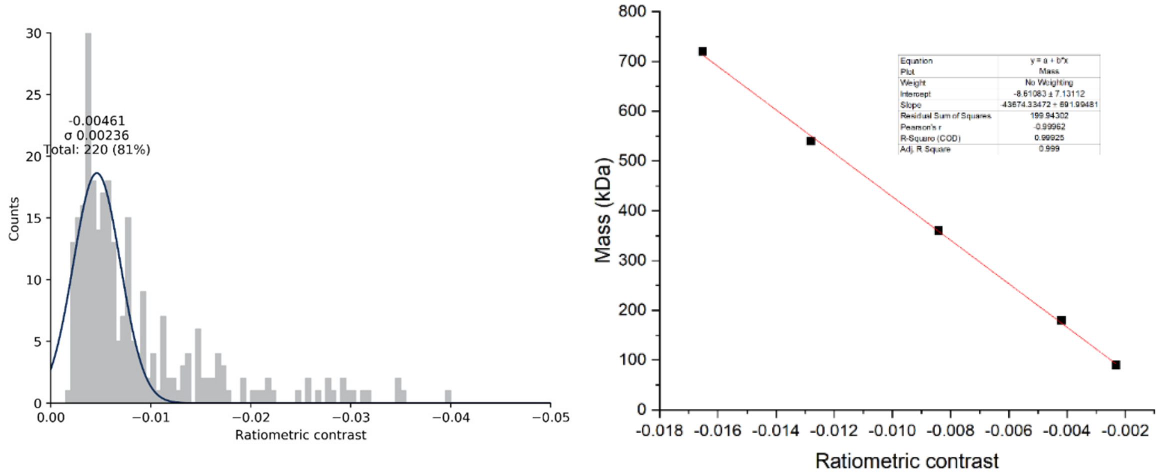

Supplementary figure 10 iSCAT meaurements on aggregates composed of 3 (left): Ratiomeric contrast distribution of supramolecular aggregates of compound **3** at pH 8 (4 μM). **(right)** Calibration curve for the conversion of ratiometric contrast to mass for a known protein oligomer solution. This calibration curve was used to estimate the average mass of the micelles of **3**.

Using the result of this calibration (b = -43674) and the average ratiometric contrast (-0.00461) an average particle mass of 201 kDa was calculated. Based on this number and the molar mass of compound **3** (629 Dalton), an aggregation number of 320 can be calculated. In addition, by assuming spherical particles with the same density as water, a diameter of 8.6 nm could be calculated, in good agreement with DLS data.

Several iSCAT images of the molecular aggregates are given on the next pages.

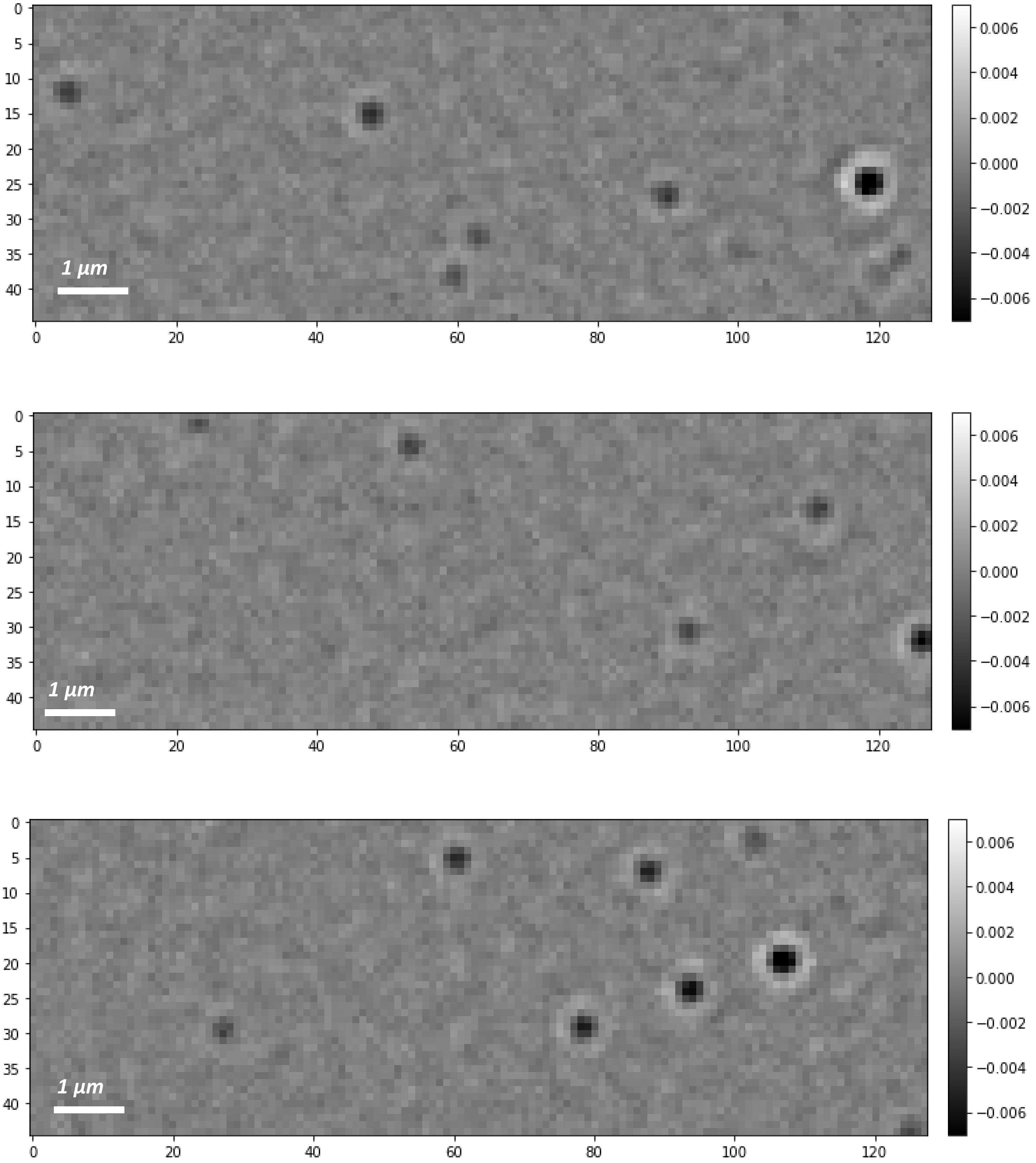
1 µm
1 µm
1 µm

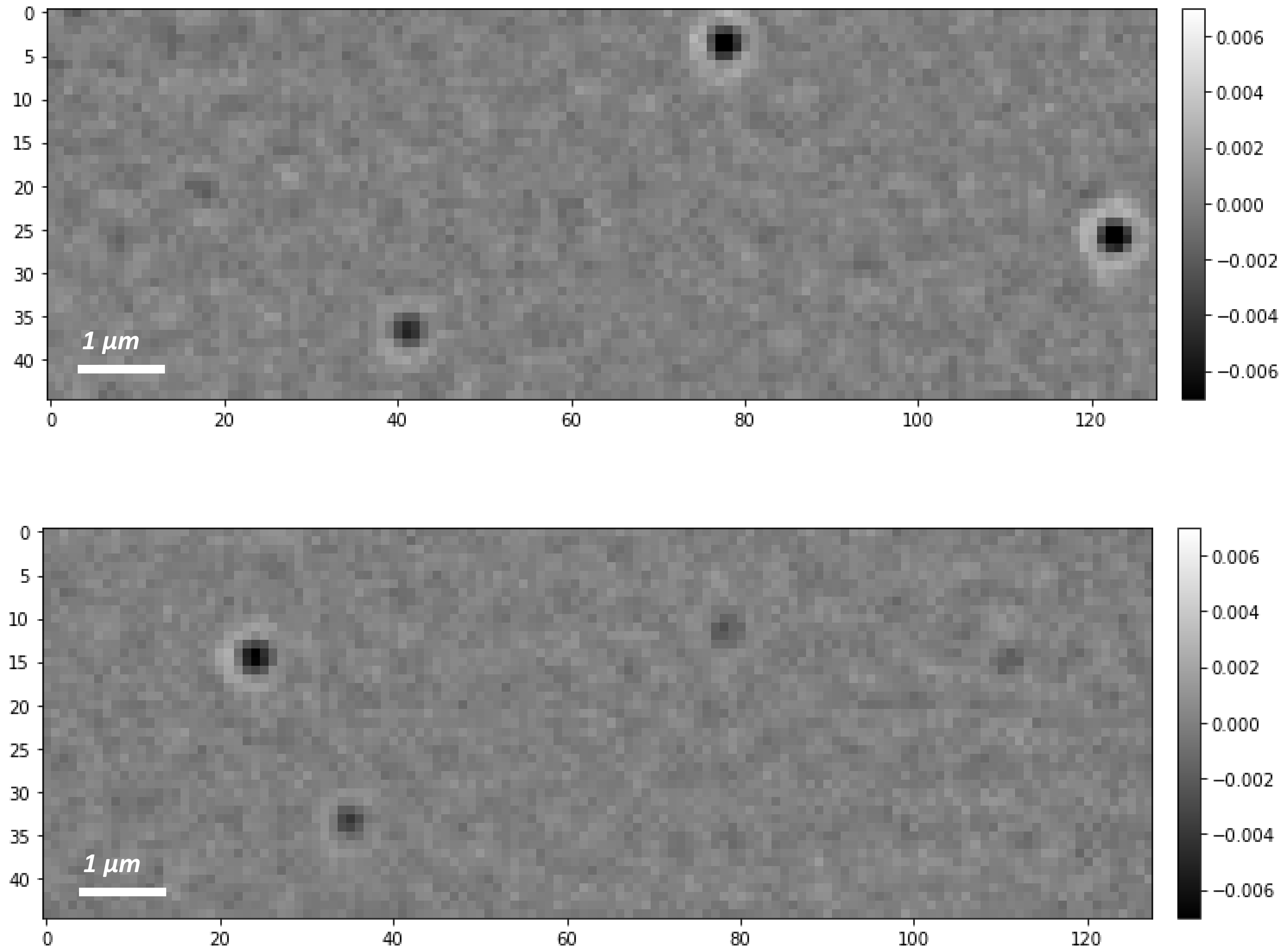
1 μm
0.006
0.004
0.002
0.000
−0.002
−0.004
−0.006
1 μm
0.006
0.004
0.002
0.000
−0.002
−0.004
−0.006

Effect of the chainlength of the alkylthiol

To investigate the effect of the chain length of the alkylthiol on the kinetics of the assembler's formation as well as the polymerization process, an additional alkyldithiol was synthesised (1,20-eicosanedithiol). We found that the surfactant synthesised from this dithiol (**3b**) aggregated in the form of vesicles instead of micelles (see Supplementary figure 11-18). While this surfactant can be synthesised, no detectable concentration could ever be measured during the biphasic (assembler) experiments. Polymerization did proceed under these conditions, but at a much slower rate than for experiments involving the (12 carbon) assembler. Seeding the experiments with neatly synthesised surfactant **3b**, resulted in fast disappearance of **3b**, while having no effect on the polymerization speed. The polymerization process for this elongated dithiol was notably slow, requiring an increase in temperature of 25 degrees (from 40 to 65°C) to achieve a same rate of polymerization.

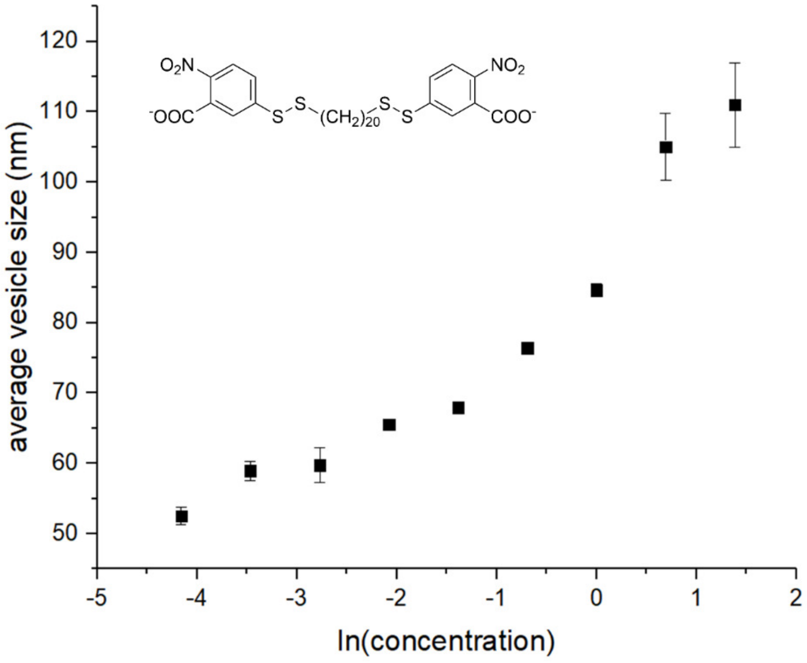

Supplementary figure 11 Effect of the concentration of 3b on its aggregate size. Average size of aggregates composed of compound **3b** as a function of its concentration determined using DLS. The positive relationship indicates aggregation in the form of vesicles. Error bars correspond to the standard deviation of the means of three experiments.

			Size (d.n...	% Intensity:	St Dev (d.n...
Z-Average (d.nm):	74.77	Peak 1:	114.9	100.0	74.16
PdI:	0.310	Peak 2:	0.000	0.0	0.000
Intercept:	0.943	Peak 3:	0.000	0.0	0.000
Result quality	Good				

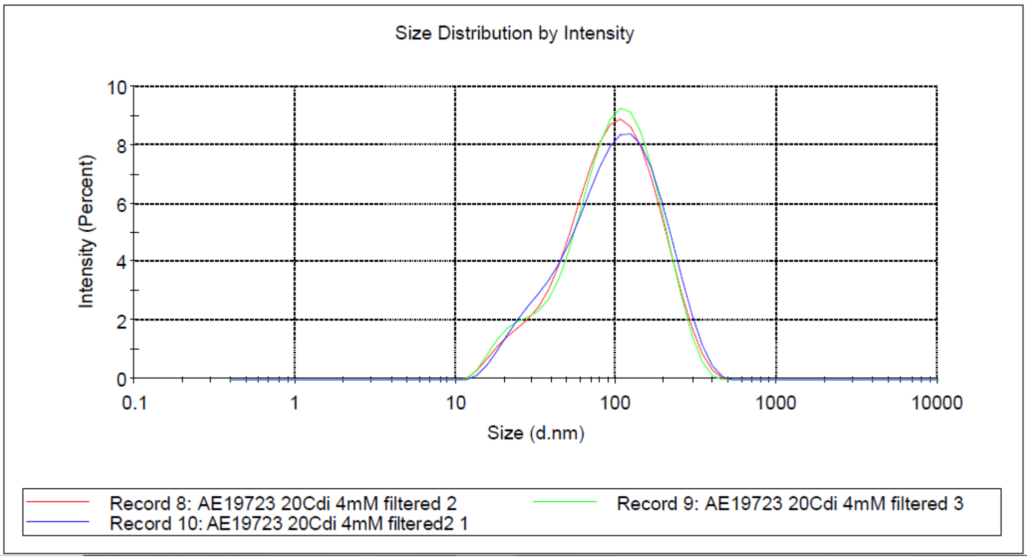

Supplementary figure 12 DLS measurement on a 4.0 mM solution of compound 3b at pH 7.5.

			Size (d.n...	% Intensity:	St Dev (d.n...
Z-Average (d.nm):	74.73	Peak 1:	98.07	96.8	54.34
PdI:	0.277	Peak 2:	3950	3.2	1101
Intercept:	0.954	Peak 3:	0.000	0.0	0.000
Result quality	Good				

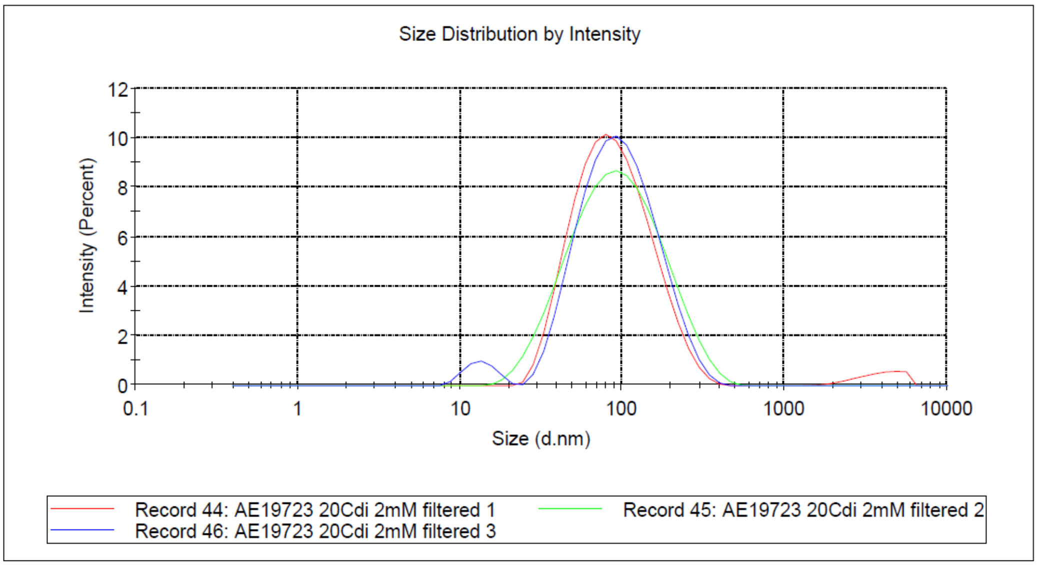

Supplementary figure 13 DLS measurement on a 2.0 mM solution of compound 3b at pH 7.5.

			Size (d.n...	% Intensity:	St Dev (d.n...
Z-Average (d.nm):	66.09	Peak 1:	83.80	100.0	33.30
PdI:	0.211	Peak 2:	0.000	0.0	0.000
Intercept:	0.959	Peak 3:	0.000	0.0	0.000
Result quality	Good				

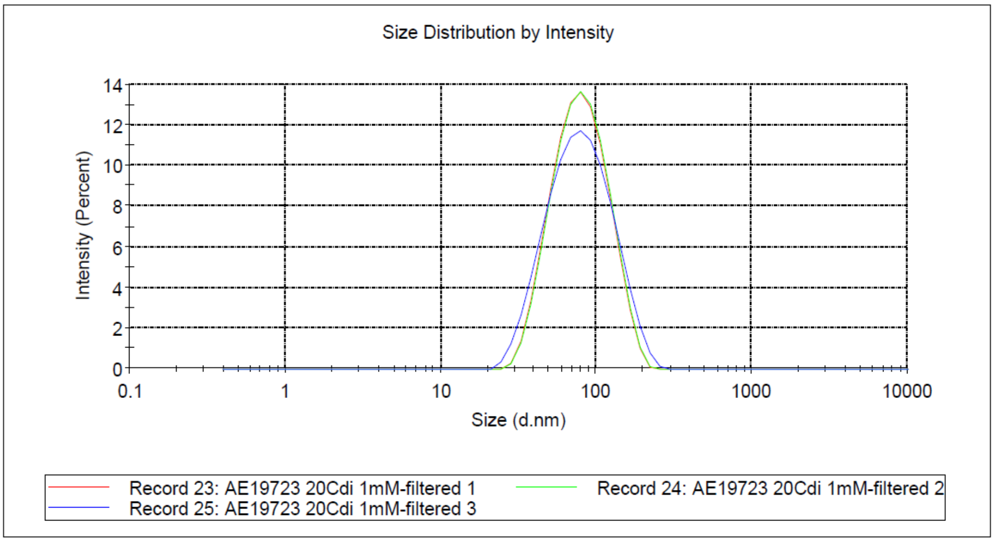

Supplementary figure 14 DLS measurement on a 1.0 mM solution of compound 3b at pH 7.5.

			Size (d.n...	% Intensity:	St Dev (d.n...
Z-Average (d.nm):	62.18	Peak 1:	75.90	100.0	32.65
PdI:	0.176	Peak 2:	0.000	0.0	0.000
Intercept:	0.936	Peak 3:	0.000	0.0	0.000
Result quality	Good				

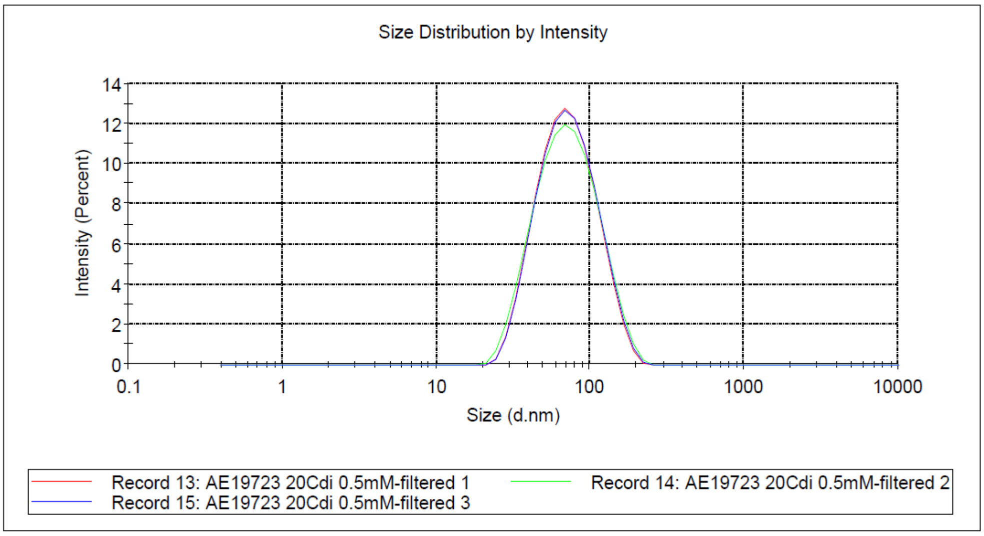

Supplementary figure 15 DLS measurement on a 0.50 mM solution of compound 3b at pH 7.5.

			Size (d.n...	% Intensity:	St Dev (d.n...
**Z-Average (d.nm):**	55.13	**Peak 1:**	67.68	100.0	32.18
**PdI:**	0.191	**Peak 2:**	0.000	0.0	0.000
**Intercept:**	0.924	**Peak 3:**	0.000	0.0	0.000
**Result quality**	**Good**				

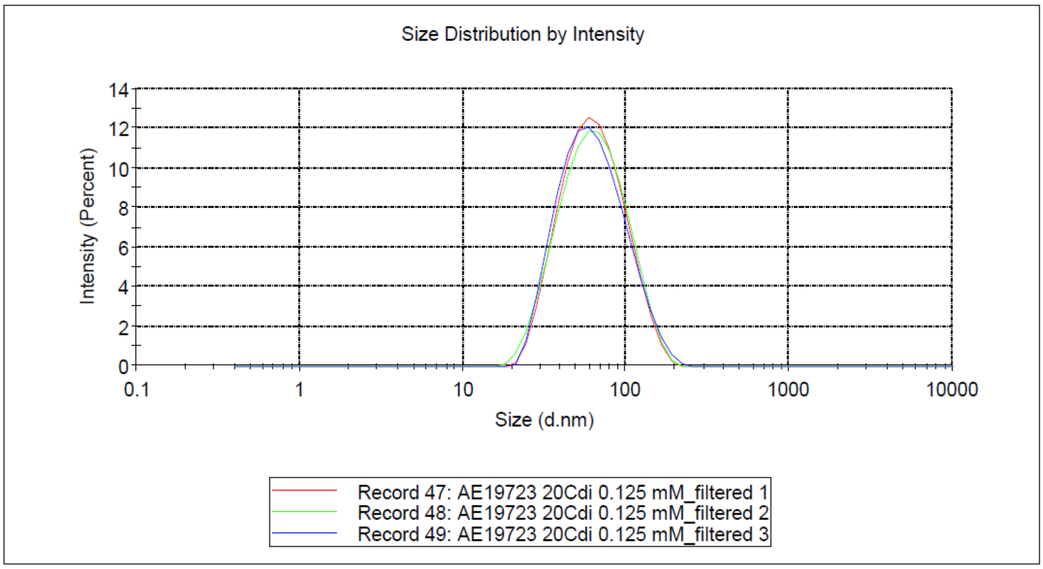

Supplementary figure 16 DLS measurement on a 0.25 mM solution of compound 3b at pH 7.5.

**Z-Average (d.nm):**	55.09	**Peak 1:**	65.72	100.0	27.15
**PdI:**	0.160	**Peak 2:**	0.000	0.0	0.000
**Intercept:**	0.921	**Peak 3:**	0.000	0.0	0.000
**Result quality**	**Good**				

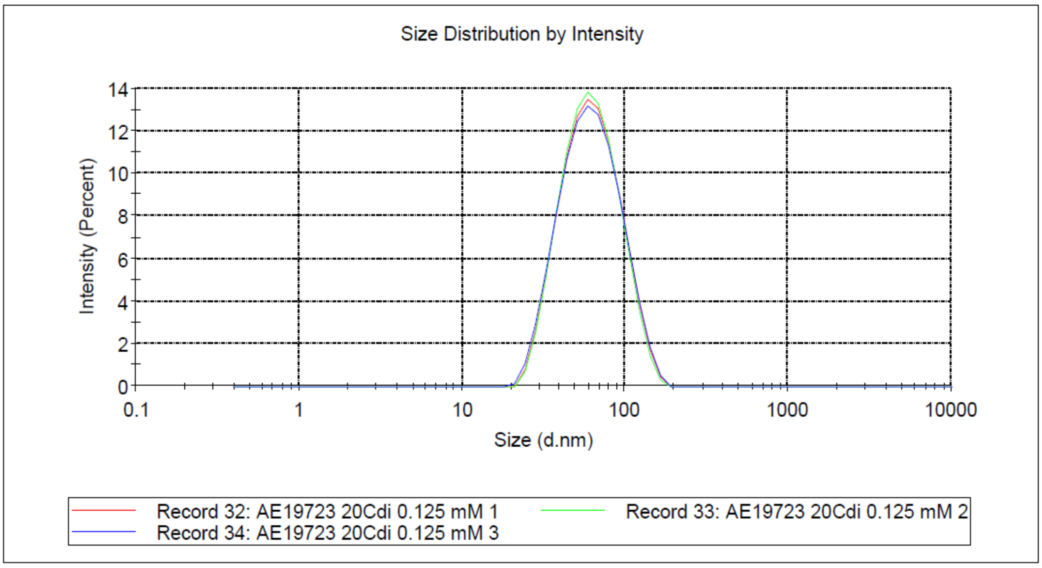

Supplementary figure 17 DLS measurement on a 0.12 mM solution of compound 3b at pH 7.5.

			Size (d.n...	% Intensity:	St Dev (d.n...
Z-Average (d.nm):	50.49	Peak 1:	62.15	98.2	28.44
PdI:	0.240	Peak 2:	4327	1.8	953.9
Intercept:	0.857	Peak 3:	0.000	0.0	0.000
Result quality	Good				

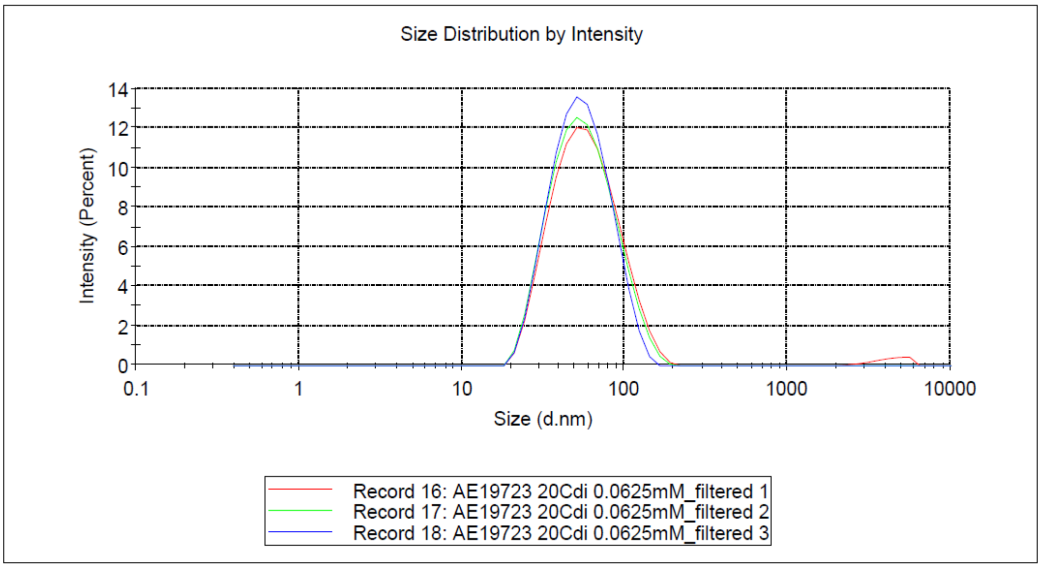

Supplementary figure 18 DLS measurement on a 0.062 mM solution of compound 3b at pH 7.5.

Synthesis of compounds

General: All commercial chemicals were purchased from Sigma-Aldrich and were used without further purification. NMR spectra were recorded on a Bruker Topspin 400 (400 MHz) spectrometer in $CDCl_3$ (unless otherwise reported). Chemical shifts are given in ppm with respect to tetramethylsilane (TMS) as internal standard. Coupling constants are reported as *J*-values in Hz. Column chromatography was carried out using Acros silica gel (43-60 µm). Both **2** and **2a** has been previously described, but were prepared using a different synthesis strategy,[2] while **3** had not been previously described. Synthesis procedures for the dithiols described here are based on the methods described by Hasegawa et al.[3] Obtained NMR spectra for previously characterized compounds were in agreement with literature.

Dodecane-1,12-diyl dimethanesulfonate (**2a**):[4]

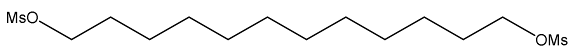

A solution of 1,12-dodecanediol (5.05g, 25 mmol) and triethylamine (7.6 mL, 55 mmol) in ethylacetate (50 mL) was cooled to 0˚C. Methanesulfonyl chloride (4.3 mL, 55mmol) was added to this solution dropwise over a period of 15 minutes. Next, the solution was stirred at room temperature for 24 hours, after which water was added. The organic phase was extracted, washed with brine and dried using $MgSO_4$. The solvent was removed under reduced pressure, and after recrystallization from diethylether yielded dodecane-1,12-diyl dimethanesulfonate as colourless crystals (6.72 g, 75%).

^{1}H NMR (400 MHz, $CDCl_3$) δ 4.21 (t, 4H, O-C*H₂*-CH₂ J=6.6 Hz), 2.99 (s, 6H, SC*H₃*) 1.74 (m, 4H, SH-CH₂-C*H₂*), 1.39 (m, 4H, C*H2*), 1.34-1.24 (m, 12H, C*H2*). ^{13}C (100 MHz, $CDCl_3$) δ 70.3, 37.5, 29.5, 29.4, 29.2, 29.1, 25.5.

Dodecane-1,12-dithiol (**2**):[2]

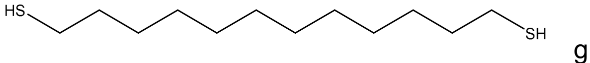

Dodecane-1,12-diyl dimethanesulfonate (6.72 g) was added to isopropanol (50 mL) together with thiourea (3.54 g, 46 mmol, 2.5 eq), after which the solution was refluxed for 24 hours. The solvent was removed to yield a white solid. The solid was suspended in 100 mL methanol and placed under nitrogen atmosphere.

A solution of NaOH (3.8 g, 92 mmol) in water (20 mL) was added and, the solution was refluxed for 2 hours. Next, the solution was acidified to pH 1, after which the product was extracted with ethylacetate, washed with brine and dried using MgSO4. The solvent was removed, after which the product was purified using column chromatography (gradient 100% hexanes → 50% DCM in hexane). Removal of the solvent yielded the title compound as colourless crystals (3.30 g, 75% yield)

^{1}H NMR (400 MHz, $CDCl_3$) δ 2.55 (q, 4H, SH-C*H₂*-CH₂ J=7.3 Hz), 1.60 (m, 4H, SH-CH₂-C*H₂*), 1.37 (m, 4H, C*H2*), 1.32 (t, 2H, S*H*, J=7.6 Hz), 1.32-1.22 (m, 12H, C*H2*). ^{13}C (100 MHz, $CDCl_3$) δ 34.2, 29.7, 29.6, 29.2, 28.5, 24.8.

5,5'-(dodecane-1,12-diylbis (disulfanediyl))bis(2-nitrobenzoic acid) (**3**):

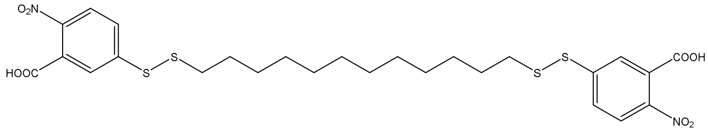

A solution of 1,12-dodecanedithiol (117 mg, 0.5 mmol), Ellman's reagent (**1**, 400 mg, 1.0 mmol) and triethylamine (0.5 mL, 3.5 mmol) in DCM (10 mL) was stirred for 1 hour, after which the solvent was removed. 1 M HCl was added (10 mL), and the product was extracted using ethylacetate (3 x 10 mL). The combined extracts were washed with brine, dried using $MgSO_4$ and the solvent removed. Purification by column chromatography (97:2:1 → 20:10:1 DCM:MeOH:AcOH) and consequent solvent removal yielded the title compound as a light yellow oil (note: NMR contains traces of acetic acid and DCM).

^{1}H NMR (400 MHz, DMSO-d_6) δ 8.01 (m, 2H, CH_{arom}), 7.92-7.83 (m, 4H, CH_{arom}), 2.84 (t, 4H, S-C*H₂*-CH₂, J=7.2 Hz), 1.59 (q, 4H, S-CH₂-C*H₂* J=7.3 Hz), 1.32 (m, 4H, C*H2*), 1.22-1.14 (m, 12H, C*H2*). ^{13}C (100 MHz, $CDCl_3$) δ 165.7, 145.7, 144.4, 128.0, 125.8, 124.8, 38.2, 28.8, 28.7, 28.5, 28.2, 27.5.

HRMS *m / z* found [M-H]- 627.0982, $C_{26}H_{31}O_8N_2S_4$ calculated 627.0969.

UPLC measurements and calibration

The concentration of UV active components of the reaction were monitored using a Waters Acquity ultra performance liquid chromatography UPLC H-Class system with photodiode array (PDA) detector. Instrument control and data processing were performed using Empower software. An Acquity UPLC BEH C18 column (130 Å, 1.7 µm, 2.1mm× 50 mm) was used. A mixture of 2O:MeCN:5% TFA in H2O with a gradient of 93:2:5 → 0:95:5 over 5 min was used as mobile phase. Peak areas were integrated at a wavelength of 330 nm. Sample preparation as well as calibration for compounds **1** and **4** has been previously described by Morrow et al.[5] The concentration calibration for compound **3** is given in figure Supplementary figure 19.

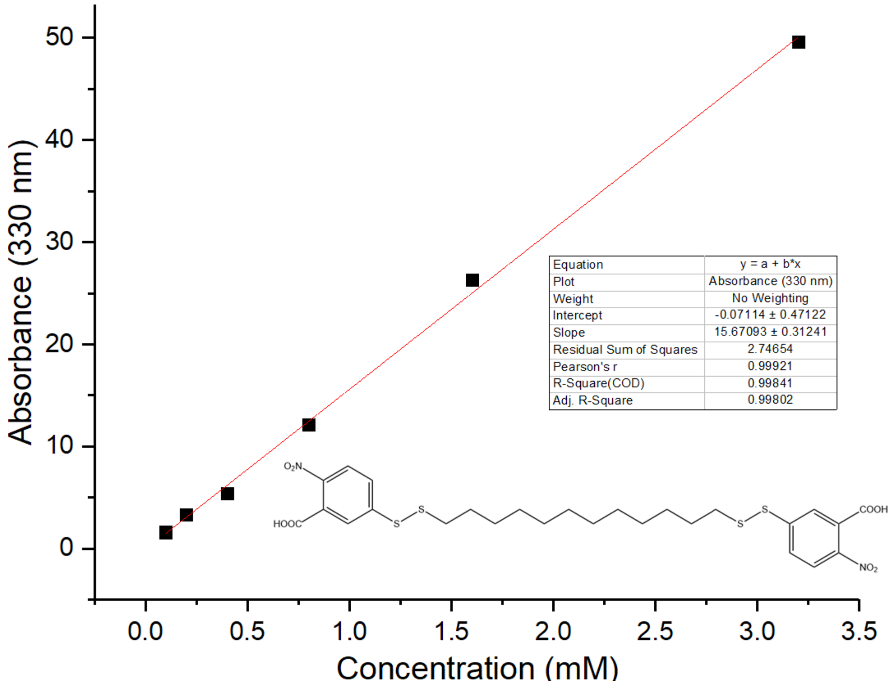

Supplementary figure 19 UPLC calibration of compound 3. The concentrations of **3** during the polymerization experiments were determined using this calibration curve.

Supplementary discussion

Polymerization mechanism

This secton describes additional experiments, carried out to provide further support for the proposed polymerization mechanism in the main manuscript.

<u>Activity of the assembler:</u>

To further establish the activity of the assembler (composed of **3**) for the production of polymers, additional experiments were carried out. In these experiments, micelles of **3** were actively removed from to reaction mixture. If the micelles of **3** act as the active polymer assembler (and not some background reaction), this should reduce the degree of polymerization. To remove the micelles, after eight hours, the reaction mixture was cooled to 0˚C after which the apolar layer solidified. The aqeous layer (containing the micelles) was removed, and replaced with a fresh buffer solution containing **1**. This action was repeated after 24 hours. After 32 hours, the apolar layer was analyzed using NMR, to determine the amount of conversion of thiol **2** to disulfide. This value was then compared to the reference experiments, to which the same cooling procedure was applied, but for which the aqeous layer (containing the micelles) was not replaced. The observed conversion to thiol was:

Experiment: 18 ± 5 %

Reference: 36 ± 2 %

Removal of the micelles of **3** thus results in a lesser degree of polymerization. Note that during the periods inbetween aqeous layer replacement, the amount of **3** had been (almost) restored to its equilibrium value. This implies that even though the amount of polymer assembly by the micelles of **3** was significantly reduced, a sufficient (average) concentration remained to explain the 18% conversion in these experiments.

<u>Polymer growth after depletion of compound **3**:</u>

Upon depletion of thiol **2**, no more surfactant **3** can be formed. Continuous destruction then results in a decrease in the concentration of **3**, until no more **3** remains. At 40˚C and pH 8.0, it takes approximately 4-5 days before **3** has completely disappeared (main manuscript, Fig 2), but polymer growth continues even after this period (main manuscript, Fig 3a/b). The remainder of the thiol groups is then converted via a different mechanism, but at a slower rate. We assume that further alkyl thiol conversion then proceeds at the interface of the water and thiol/disulfide layers, since no significant amount of any surfactant can be detected by UPLC any longer.

Supplementary figures

NMR spectra

NMR spectra were recorded on a Bruker Topspin 400 (400 MHz) spectrometer in CDCl3. Chemical shifts are given in ppm with respect to tetramethylsilane (TMS) as internal standard.

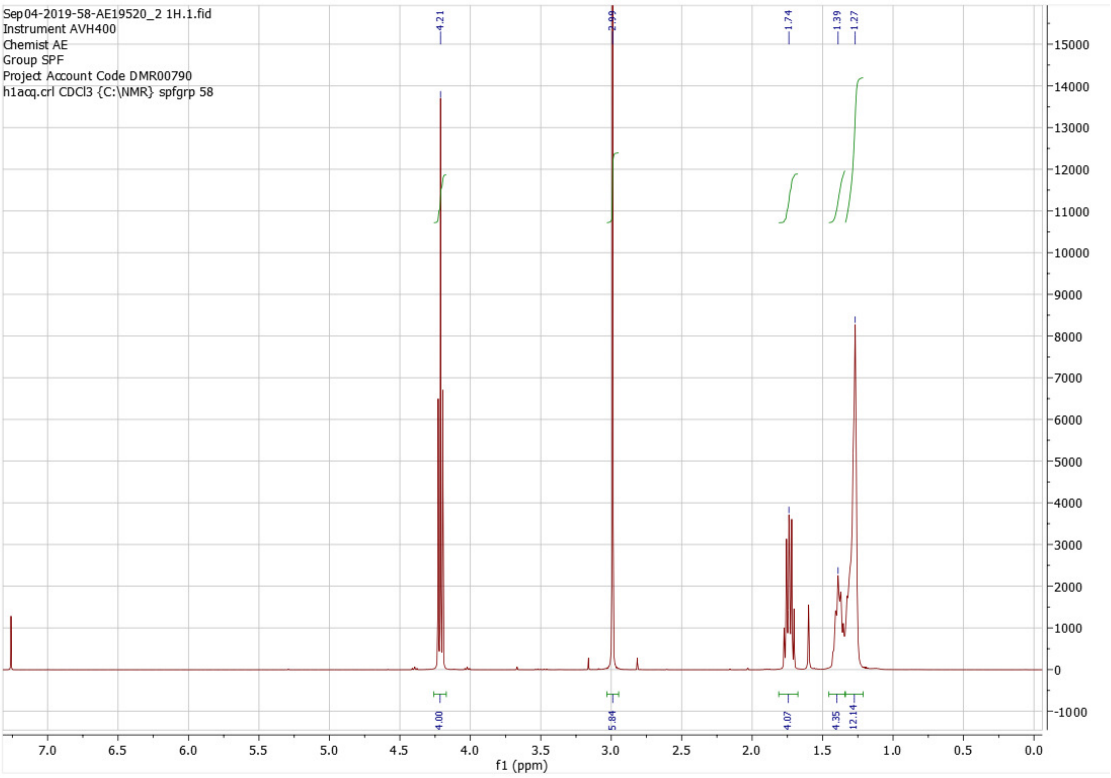

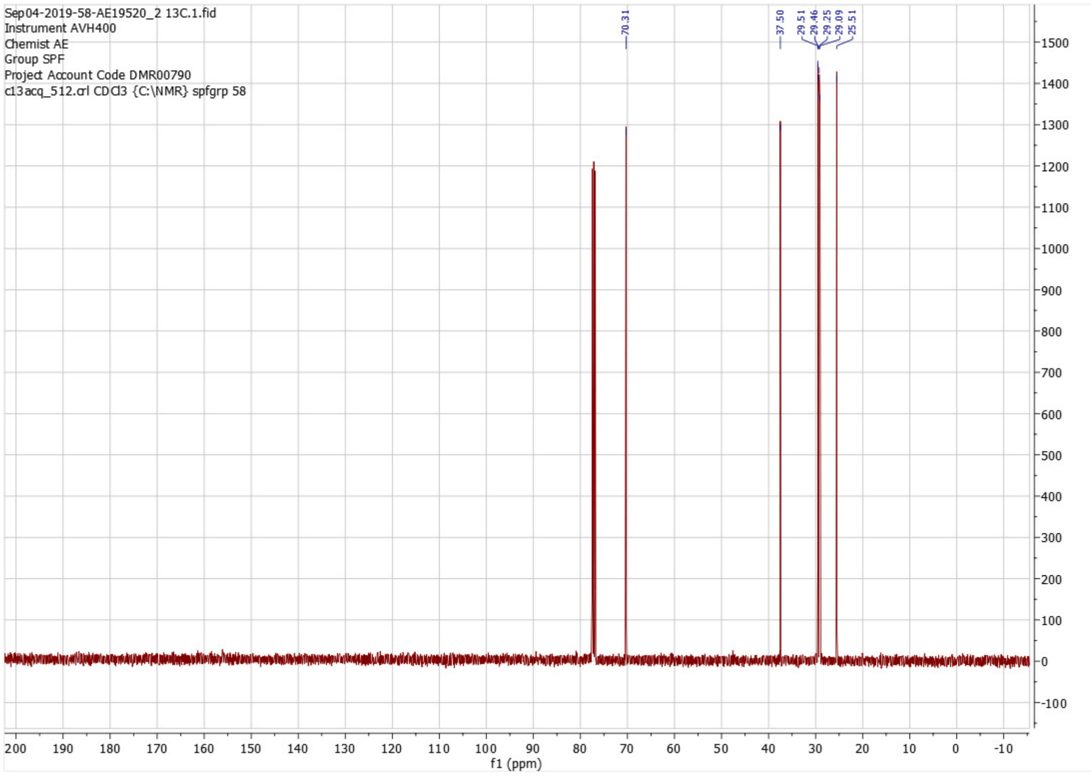

Supplementary figure 20 ^{1}H NMR and ^{13}C NMR spectra of dodecane-1,12-diyl dimethanesulfonate (2a)

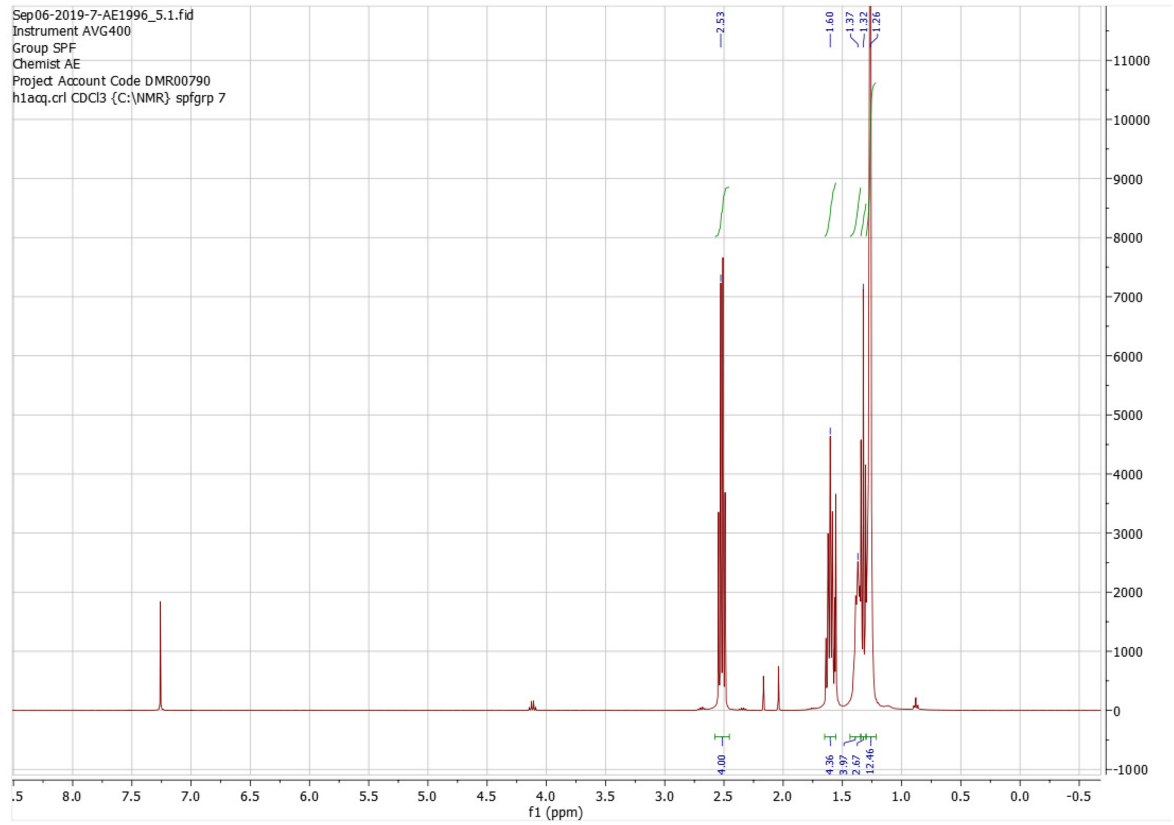

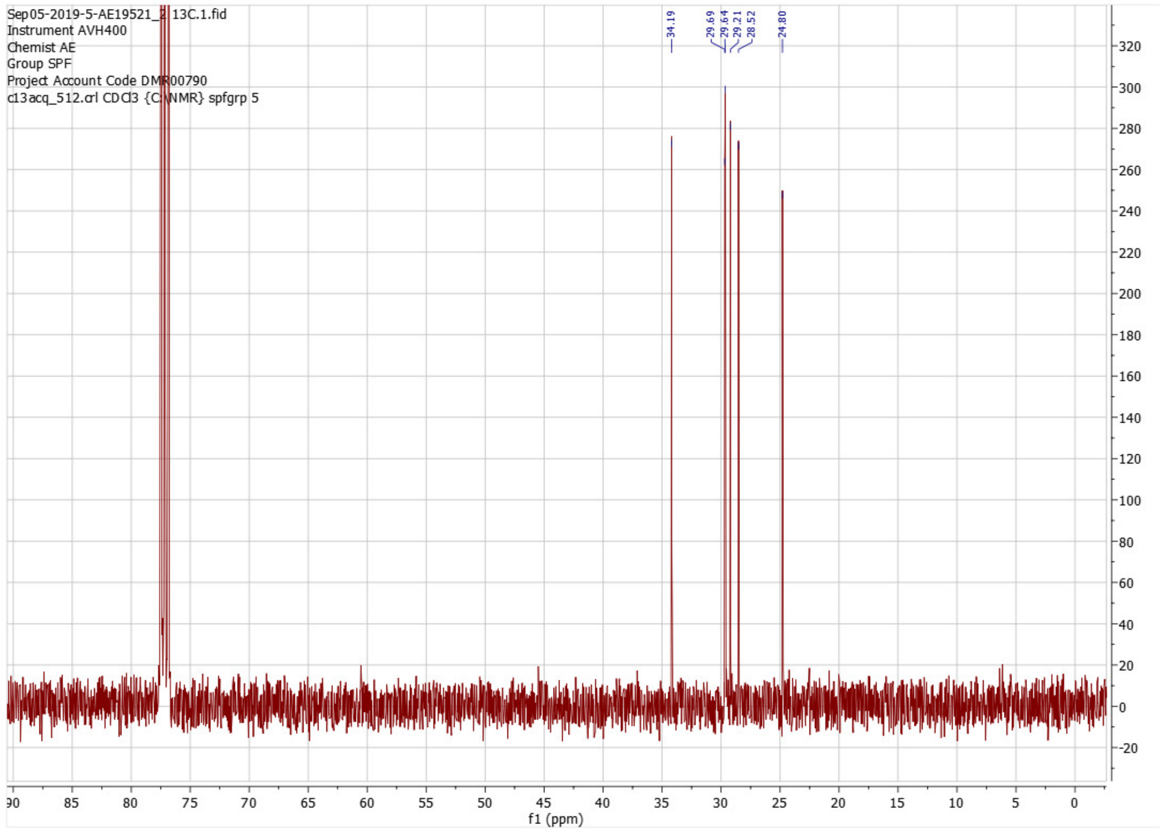

Supplementary figure 21 ^{1}H NMR and ^{13}C NMR spectra of Dodecane-1,12-dithiol (2)

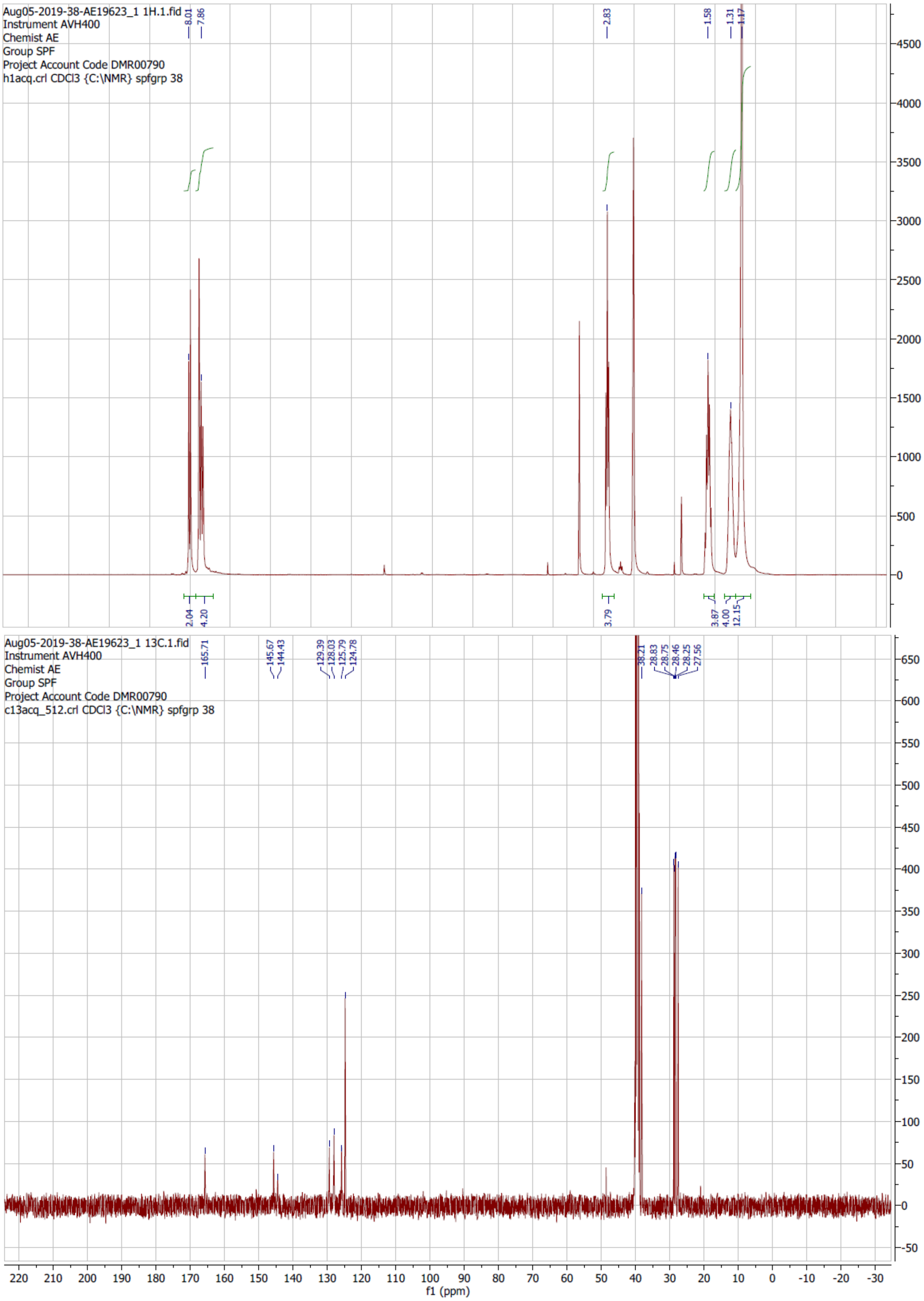

Supplementary figure 22 ^{1}H NMR and ^{13}C NMR spectra of 5,5'-(dodecane-1,12-diylbis (disulfanediyl))bis(2-nitrobenzoic acid) (3)

NMR analysis of polymer size:

The average chain length of the produced polymers can be determined based on the thiol to disulfide ratio (assuming negligible cyclized products). Since the S**C*H₂*** proton signals of the thiol and disulfide are sufficiently separated in ^{1}H NMR, the ratio of these peaks was used to determine the average chainlength:

Disulfide: δ 2.68 (t, J= 7.5 Hz)

Thiol: δ 2.52 (q, J= 7.3 Hz)

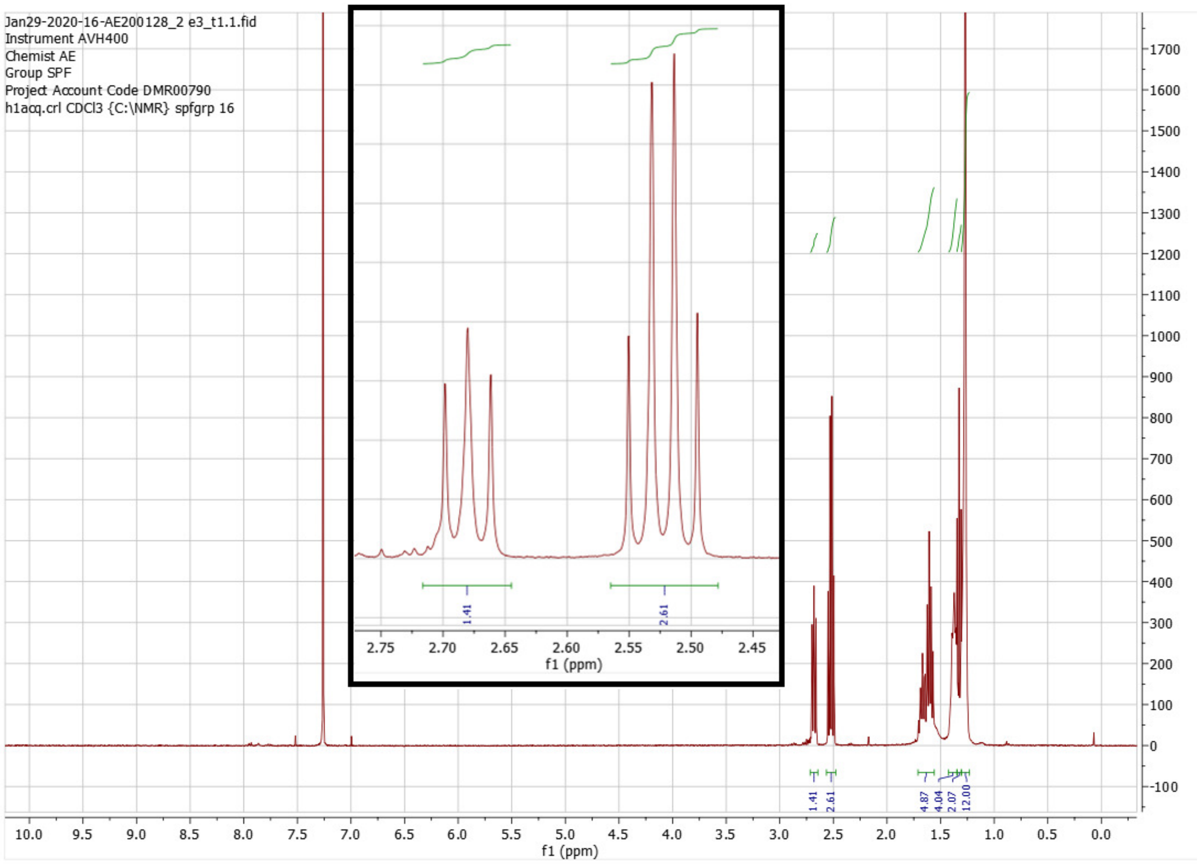

Supplementary figure 23 ^{1}H NMR of disulfide polymers made from 2.

Supplementary references

1. Young, G. et al. Quantitative mass imaging of single biological macromolecules. *Science* **360**, 423-427 (2018).
2. Kushmerick, J.G. et al. Effect of bond-length alternation in molecular wires. *J. Am. Chem. Soc.* **124**, 10654-10655 (2002).
3. Hasegawa, J. et al. The application of phenylmethanethiol and benzenethiol derivatives as odorless organosulfur reagents in the synthesis of thiosugars and thioglycosides. *Carbohydr. Res.* **340**, 2360-2368 (2005).
4. Bigot, S., Kebir, N., Plasseraud, L. & Burel, F. Organocatalytic synthesis of new telechelic polycarbonates and study of their chemical reactivity. *Polymer* **66**, 127-134 (2015).
5. Morrow, S.M., Colomer, I. & Fletcher, S.P. A chemically fuelled self-replicator. *Nat. Commun.* **10** (2019).
